# Sera from Children with Autism Induce Autistic Features Which Can Be Rescued with a CNTF Small Peptide Mimetic in Rats

**DOI:** 10.1371/journal.pone.0118627

**Published:** 2015-03-13

**Authors:** Syed Faraz Kazim, Maria del Carmen Cardenas-Aguayo, Mohammad Arif, Julie Blanchard, Fatima Fayyaz, Inge Grundke-Iqbal, Khalid Iqbal

**Affiliations:** 1 Inge Grundke-Iqbal Research Floor, Department of Neurochemistry, New York State Institute for Basic Research in Developmental Disabilities (NYSIBR), Staten Island, New York, United States of America; 2 Neural and Behavioral Science Graduate Program, State University of New York (SUNY) Downstate Medical Center, Brooklyn, New York, United States of America; 3 SUNY Downstate/NYSIBR Center for Developmental Neuroscience (CDN), Staten Island, New York, United States of America; Univeristy of Miami, UNITED STATES

## Abstract

Autism is a neurodevelopmental disorder characterized clinically by impairments in social interaction and verbal and non-verbal communication skills as well as restricted interests and repetitive behavior. It has been hypothesized that altered brain environment including an imbalance in neurotrophic support during early development contributes to the pathophysiology of autism. Here we report that sera from children with autism which exhibited abnormal levels of various neurotrophic factors induced cell death and oxidative stress in mouse primary cultured cortical neurons. The effects of sera from autistic children were rescued by pre-treatment with a ciliary neurotrophic factor (CNTF) small peptide mimetic, Peptide 6 (P6), which was previously shown to exert its neuroprotective effect by modulating CNTF/JAK/STAT pathway and LIF signaling and by enhancing brain derived neurotrophic factor (BDNF) expression. Similar neurotoxic effects and neuroinflammation were observed in young Wistar rats injected intracerebroventricularly with autism sera within hours after birth. The autism sera injected rats demonstrated developmental delay and deficits in social communication, interaction, and novelty. Both the neurobiological changes and the behavioral autistic phenotype were ameliorated by P6 treatment. These findings implicate the involvement of neurotrophic imbalance during early brain development in the pathophysiology of autism and a proof of principle of P6 as a potential therapeutic strategy for autism.

## Introduction

Autism and autism spectrum disorders (ASDs) are neurodevelopmental disorders of as yet unknown etiology characterized clinically by a behavioral phenotype comprising of impaired social interaction, absence or delay in language, and repetitive, stereotyped purposeless behavior [1–3]. The onset of symptoms usually occurs after 3 years of life [4,5]. The prevalence of autism has increased dramatically over the last decade with most recent Center for Disease Control and Prevention (CDC) estimates suggesting that ASDs affect 1 in 88 children in U.S. with a five-fold higher occurrence in boys as compared to girls [6]. Even though the exact etiology of autism is as yet not precisely elucidated, existing scientific literature suggests a multifactorial etiopathogenesis encompassing genetic, environmental, and immunological factors, neurotrophic dysregulation, and an increased susceptibility to oxidative stress [1,7,8].

A consistent phenomenon reported in scientific literature on autism cases is an accelerated brain growth during early development followed by slowed brain growth and decreased neuronal number and size and less dendritic branching in various brain regions such as cerebellum, hippocampus, and amygdala [9–17]. These findings point towards an abnormality in regulatory mechanisms that govern growth and differentiation of central nervous system leading to an imbalance in neuronal and synaptic formation and pruning [7,18]. One of the most prominent factors in neurogenesis, neuronal proliferation, differentiation, and pruning in normal brain development is the microenvironment provided by various neurotrophic factors [7]. A dysregulation of neurotrophic factors can be a major cause of abnormalities in neurogenesis, neuronal migration and differentiation, synaptic connectivity and maturation, and neuronal and synaptic pruning leading to deficits in social behavior and cognition observed in autism. Alterations in the levels of neurotrophic factors in the brain, cerebrospinal fluid (CSF), and blood of individuals with autism have been reported extensively (for review, [1,3,7]). A main cause of dysregulation of neurotrophic factors in autism might be oxidative stress during prenatal and early development which is a widely implicated in the pathogenesis of autism [8]. For example, increased oxidative stress has been shown to block ciliary neurotrophic factor (CNTF) activity in neurons which is essential for neuronal survival and maintenance [19–21]. On a similar note, serum levels of brain-derived neurotrophic factor (BDNF) have been linked to oxidative stress in ASDs [22]. Previously, cerebrolysin, a peptidergic neurotrophic preparation [23] which has been shown to protect chicken cortical neurons from neurodegeneration in an iron-induced oxidative stress model [24] and to enhance dentate gyrus neurogenesis and associated memory in normal adult rats [25] was found to improve expressive and receptive speech and fine motor performance in 17 out of 19 children with autism [26]. Targeting the neurotrophic abnormalities in autism can, thus, serve as potential therapeutic approach.

The therapeutic usage of neurotrophic factors such as BDNF and CNTF has been limited primarily because full-length neurotrophic factor molecules poorly reach the central nervous system after peripheral administration and have short plasma half-lives [27–29]. Besides, recombinant CNTF was shown to cause anorexia, skeletal muscle loss, hyperalgesia, severe cramps, and muscle pain in human clinical trials [30]. Previously, by mapping the biologically active region of human CNTF, we generated an 11-mer peptide, Peptide 6 (P6), which is blood-brain barrier (BBB) permeable, has a plasma half-life of over 6 hr, and does not cause adverse effects associated with the full-length protein in mice or rats [31–35]. This CNTF derived small peptide mimetic is shown to exert beneficial effect on neurogenesis, neuronal and synaptic plasticity, and cognition via inhibition of LIF signaling pathway and elevation of BDNF level by increasing its transcription [31,35].

In the present study, we show that (i) sera from children with autism cause neurodegeneration and increased oxidative stress in embryonic day 18 mouse primary neuronal cultures; (ii) intracerebroventricular injection of autistic sera within hours after birth produces characteristic autistic behavioral phenotype in young rats; and (iii) pre-treatment with P6 is neuroprotective to autistic sera-induced changes both *in vitro* in primary neuronal cultures and *in vivo* in rats.

## Materials and Methods

### Sera from children with autism and from healthy controls

Studies carried out on human sera described in the present report were approved by the Institutional Review Board (IRB) of the New York State Institute for Basic Research in Developmental Disabilities in accordance with the NIH Guide and Federal Wide Assurance FWA00006105. As all study participants were minors, the written informed consent was obtained from caretakers on forms approved by our institutional IRB. Table 1 summarizes the general clinical profiles of 22 pairs of children with autism and healthy controls whose sera were screened in *in vitro* studies and Table 2 provides details of the 3 pairs of these autism and control subjects whose sera were used for further *in vitro* and *in vivo* investigations. The diagnosis of autism was made using Autism Diagnostic Observation Schedule-Generic (ADOS-G) and the Diagnostic and Statistical Manual of Mental Disorders, 4^th^ edition (DSM-IV). Further confirmation of the diagnosis was performed with Autism Diagnostic Interview-Revised (ADI-R), an abridged version of ADI administered through interviewing the parents (Tables 1 and 2). Additional characterization of the subjects was carried out by Vineland Adaptive Behavior Scale (VABS). Blood samples were collected from children in families belonging to the same population based in the New York City (NYC) area and expectedly exposed to similar profile of external environmental factors e.g. air pollution and water contamination. Additional differences owing to variable child care, dietary patterns, inherent household customs and preferences, and various other non-specific environmental factors that may play some role in the development of autism but have not yet been assigned a definitive role were not taken into consideration. The level of IQ was not used as a selection criterion for donors as it is not a definitive diagnostic tool for autism. Control samples were collected from normal children who had siblings with autism but were not related to the probands evaluated in the current study. All autism and control subjects belonged to the ethnic group white. The study subjects did not have any significant history of seizures or gastrointestinal problems, none had received a concomitant diagnosis of fragile X syndrome or Rett syndrome, and none were on antidepressants, neuroleptics, seizure medications, or stimulants. All sera were stored as coded anonymous samples at -80°C. Immediately before experiments, sera were thawed once and used for *in vitro* and *in vivo* studies as indicated.

**Table 1:** Characterization of serum donors initially screened

*Diagnosis (DSM-IV; ADOS-G)*	*Autism*	*Control*
**Number of donors (M/F)**	22 (15/7)	22 (13/9)
**Mean Age (±S.E.M.**)	4.96±0.11	5.17±0.195
**Age range**	4.1–6	3.76–6.4
***Autism Diagnostic Interview-Revised Scale (ADI-R)***		
**QA in reciprocal social interaction**	17.45±1.47 (7–28)^a^	N/A
**QA in communication**	12.14±0.58 (7–20)	N/A
**Restricted, repetitive and stereotyped behavior**	5.09±0.41 (3–12)	N/A
***Vineland Adaptive Behavior Scale (VABS)***		
**Communication**	58.86±3.9 (40–106)	N/A
**Daily living skills**	59±3.01 (36–104)	N/A
**Socialization**	58.14±1.81 (49–78)	N/A
**Motor**	61.41±2.93 (41–93)	N/A

^a^ Range of the results

Abbreviations: ADOS-G; Autism Diagnostic Observation Schedule-Generic; DSM-IV, Diagnostic and Statistical Manual of Mental disorders, 4^th^ edition; N/A, Not applicable

**Table 2:** Description of sera used for final ***in vitro*** and ***in vivo*** analyses

*Diagnosis* ^*a*^	*Age*	*Gender*	*ADIQRS*	*ADICOM*	*ADIREPST*	*CSS*	*DSS*	*SSS*	*MSS*
**Autism**	5.11	M	16	14	5	57	58	57	59
**Autism**	4.8	M	15	13	8	61	63	58	56
**Autism**	4.6	M	25	12	4	73	65	63	61
**Control**	5	M	N/A	N/A	N/A	N/A	N/A	N/A	N/A
**Control**	5.4	M	N/A	N/A	N/A	N/A	N/A	N/A	N/A
**Control**	4.8	M	N/A	N/A	N/A	N/A	N/A	N/A	N/A

^a^ Diagnosis based on DSM-IV; ADOS-G

Abbreviations: ADIQRS, ADI-R QA in reciprocal social interaction; ADICOM, ADI-R QA in communication; ADIREPST, ADI-R restricted, repetitive and stereotyped behavior; CSS, VABS communication; DSS, VABS daily living skills; SSS, VABS socialization; MSS, VABS motor; N/A, Not applicable

### Design and synthesis of P6

P6 which corresponds to amino acid residues 146–156 of human CNTF was identified as an active region of this neurotrophic factor by epitope mapping of neutralizing antibodies to CNTF, as described before [31–33]. The peptide was synthesized using solid phase peptide synthesis (SPSS) methods, purified by reverse phase HPLC to >96% purity, lyophilized, and characterized via HPLC, NMR, and ESI-MS [31–33].

### In vitro studies

**Study outline**. The effects of treatment with the sera from autistic and control children and of P6 were evaluated in the *in vitro* studies using primary cortical neuronal cell cultures from embryonic day 18 (E18) mouse cortex. The cultured neurons were treated on 4^th^ day in vitro (DIV4, 72 hours after seeding) and their morphology was analyzed on DIV7 (72 hours after treatment). Subsequently, cell death and viability, and oxidative stress were analyzed.

**Primary neuronal cultures**. Primary cortical neuronal cell cultures were prepared from E18 C57BL/6 mice cortex. The procedure for the primary culture was carried out as previously reported [36–38]. Briefly, C57BL/6 time pregnant E18 female mice from Charles River labs were anesthetized and killed by cervical dislocation. All studies were performed in accordance with the recommendations in the Guide for the Care and Use of Laboratory Animals of the National Institutes of Health (NIH). The protocol was approved by the Institutional Animal Care and Use Committee (IACUC) of the New York State Institute for Basic Research in Developmental Disabilities (Protocol no. 199). Embryos were removed and placed in cold hibernate A (Brain bits, Springfield, IL, USA), and all following steps were performed in ice-cold hibernate A, using the stereoscopic (dissection) microscope placed in a laminar flow hood. Fetal brains were removed carefully; cerebral cortex was separated, and was dissected and cut into small pieces using microsurgical scissors. The cut tissue was transferred with number 5 forceps to 15 ml tubes containing 0.1% trypsin in versene (Invitrogen Life Technologies, Grand Island, NY, USA) and incubated for 15 min at 37°C followed by inactivation with 10% fetal bovine serum (FBS) in Neurobasal complete medium (Neurobasal Medium supplemented with 2x B-27, 0.3% glutamine, and penicillin/streptomycin 0.1 mg/ml and 0.1 U/ml respectively). After 72 hours, the medium was replaced and supplemented with fresh medium with or without autism or control serum± P6 as described below. All medium components were purchased from Invitrogen Grand Island, NY, USA. Cells were maintained in an incubator at 37°C at 5% CO_2_/95% atmospheric air. For recovering the protein, cells were seeded in 6-well plates precoated overnight with 50 μg/ml poly-D-lysine (Sigma-Aldrich, st. Louis, MO, USA) at a density of 1x10^6^ cells/well. For immunocytochemistry, LDH and oxidative stress assays, cells were seeded onto 8-well chambers or 96-well plates (precoated with poly-D-lysine) at a density of 8x10^4^ cells/well or 7x10^4^ cells/well in 300 or 100 μl Defined Medium, respectively.

**Treatment of cultured neurons with sera with or without P6**. The cells were cultured for 72 hours prior to beginning of the treatment with a serum alone or serum with P6 or vehicle. Initially, the effect of 22 pairs of autism/control sera were evaluated in 3 separate set of primary cultures. Based on these experiments, 3 pairs of sera with consistent marked effect on neuronal morphology were selected for further experiments. Initially different concentrations of the sera in culture medium (0.1%, 0.2%, 0.5%, and 1%) were evaluated and based on these analyses, a final concentration of 0.2% was chosen for subsequent experiments. Similarly, different concentrations of P6 (0.0005 μM, 0.005 μM, 0.05 μM, and 1 μM) were evaluated.

To study the cytotoxic effects of autism sera and the potential rescue with P6, 72 hours after seeding the cells, the culture medium was replaced with fresh medium containing different concentrations of P6. The P6 was dissolved in water from which necessary amount was added directly to the culture medium to achieve the desired final concentration. Three hours after the pre-treatment with P6, sera from autistic or control children were added to the culture medium already containing P6 to achieve a final concentration of sera to be 0.2%. Few wells in each plate or chamber were left vehicle treated only to serve as controls. The treatment continued for a total of 72 hours after which light microscopic evaluation of cultured neurons was performed and low and high magnification images were captured using Nikon digital camera system for digital sight, DS-Fi 1 coupled with Nikon Labophot microscope. After a total of 6 days-in-vitro, immunohistochemical analysis and LDH and oxidative stress assays were performed in different set of experiments.

**LDH assay for cell death and cell viability**. Cell death and cell viability were analyzed using the LDH cytotoxicity assay kit (Promega, Madison, WI, USA), following manufacturer’s instructions. Cell death (LDH release at OD 490 nm) and cell viability (percent of control) were plotted separately.

**Oxidative stress assays**. Dichlorofluorescein (DCF) fluorescence assay for evaluating the generation of free radicals was used to determine the intracellular production of reactive oxygen species as described previously [39]. Briefly, the primary neuronal cells were treated with the cell permeable 2, 7-dichlorofluorescein diacetate, DCFH-DA (Sigma, St. Louis, MO, USA) which is converted into 2', 7'-dichlorofluorescein. The 2', 7'-dichlorofluorescein interacts with intracellular peroxides to form a highly fluorescent compound. The medium was removed three days after serum with or without P6 treatment and cells were washed with Hank’s Balanced Solution, HBSS (Invitrogen, Camarillo, CA, USA). The cells were incubated with DCFH-DA (10 μM) for 30 min and then washed with HBSS solution two times. DCF fluorescence was quantified (excitation wave length = 485 nm, emission wave length = 530 nm) using a fluorescence multi well plate reader (Spectra Max M5, Molecular Devices, Sunnyvale, CA, USA).

Lipid peroxidation was assessed by determining the level of thiobarbituric acid reactive substance (TBARS) in primary neuronal cell lysates as described previously [40] with minor modification. Cultured neurons were lysed in lysis buffer (50 mM HEPES, pH 7.5, 1% Triton X-100, 50 mM NaCl, 5 mM EGTA, 50 mM sodium fluoride, 20 mM sodium pyrophosphate, 1 mM sodium vanadate, 2 mM PMSF, and 8 mM diisopropylfluorophosphate) containing 0.05% butylated hydroxytoluene (BHT). The 100 μl of cell lysate was added to 200 μl ice-cold 10% trichloroacetic acid (TCA) on ice for 15 min to precipitate protein. Precipitated samples were centrifuged at 2200x*g* for 15 min at 4°C. Supernatants were mixed with an equal volume of 0.67% thiobarbituric acid and then boiled for 10 min. Once cooled, the absorbance was read at wave length 532 nm on an absorbance plate reader (Spectra Max M5, Molecular Devices, Sunnyvale, CA, USA). Malonyldialdehyde (MDA, an end product of peroxidation of polyunsaturated fatty acids and related esters and a marker of lipid peroxidation) content was calculated using a molecular extinction coefficient for MDA of 2.56 x10^5^.

**Immunocytochemistry of cultured neurons for β-III-tubulin staining**. After 3 days of treatment, cells seeded in 8-well chambers were fixed in 4% paraformaldehyde (Electron Microscopy Sciences, PA, USA) for 30 min at room temperature, and then washed two times in PBS for storage at 4°C prior to staining. Cells were permeabilized in 0.05% Triton-X-100 in PBS for 20 min at 25°C, washed in PBS 3x10min, and then incubated in blocking buffer (1% BSA w/v, 0.2% Triton- X-100 v/v in PBS) for 45 min at 25°C. The cells were then incubated with rabbit polyclonal anti-Tuj-1, β-III-tubulin (1:200, Covance, Emeryville, CA, USA) antibody in blocking buffer at 4°C overnight. The cells were washed three times for 10 min in PBS and then incubated with fluorescently-labeled CY3-conjugated goat anti-rabbit secondary antibody (1:500, Jackson Laboratory, Maine, USA) diluted in blocking buffer for 2 h at 25°C in the dark. The cells were washed 3 x 10 min in PBS and 24x60 mm cover glass (Brain Research Laboratories, Newton, MA, USA) was mounted with Vectashield anti-fade mounting medium (Vector Laboratories Inc., Burlingame, CA, USA) and sealed with nail polish. The slides were examined using 20x and 40x objectives of a Nikon 90i fluorescent microscope equipped with Nikon C1 three-laser confocal system and a Nikon DS U1 digital camera, and analyzed with EZ-C1 Viewer Image software, Version 6.0.

**Western blots of human serum samples for neurotrophic factors levels**. For Western blots to evaluate the levels of various neurotrophic factors in sera samples, the serum samples were diluted in loading buffer and loaded as appropriate assuming normal human serum protein concentration to be ~80 μg/μL. 10% or 12.5% SDS-PAGE gels were employed followed by transfer of separated proteins on 0.45 μm PVDF membranes (Pall, Pensacola, FL, USA) for Western blots. The following primary antibodies were used at the indicated dilutions: rabbit polyclonal anti-CNTF, FL-200 (1:500, Santa Cruz Biotechnology, Santa Cruz, CA, USA); rabbit polyclonal anti- BDNF, N-20 (1:1000, Santa Cruz Biotechnology, Santa Cruz, CA, USA); goat polyclonal anti-LIF, N-18 (1:500, Santa Cruz Biotechnology, Santa Cruz, CA, USA); rabbit polyclonal anti-NGF, M-20 (1:1000, Santa Cruz Biotechnology, Santa Cruz, CA, USA); and rabbit polyclonal anti-FGF2, 147 (1:500, Santa Cruz Biotechnology, Santa Cruz, CA, USA). Blots were blocked for 1 hr at 37°C in TBST (0.05% Tween 20 in TBS) containing 5% w/v blotting grade dry milk (Bio-Rad, Hercules, CA, USA), incubated in primary antibody in blocking buffer overnight at 4°C, washed 3 times for 10 min in TBST at room temperature, followed by incubation with secondary antibody i.e. peroxidase-conjugated anti-rabbit or anti goat IgG (Jackson ImmunoResearch Laboratories, West Grove, PA, USA) diluted in blocking buffer. Blots were washed 3x 10 min in TBST and immunoreactive protein bands were visualized with enhanced chemiluminescence (ECL) reagents (Pierce, Rockford, IL, USA). The ECL films of the blots were scanned and analyzed using Multi Gauge software version 3.0 (Fujifilm, Tokyo, Japan). For loading control, the blots were developed with rabbit polyclonal antibody to GAPDH (1:1000, Santa Cruz Biotechnology, Santa Cruz, CA, USA). For quantification of different protein levels, each immunoreactive band was normalized to it's corresponding GAPDH band.

### *In vivo* studies in rats

**Study outline**. To study the effect of sera and P6 in the *in vivo* setting, new born Wistar rat pups (within 24 hours of birth) were injected intracerebroventricularly with sera (final concentration ~2%) from autism or control children with or without P6 (final concentration ~20 nM). The same 3 pairs of sera which showed consistent marked effects in *in vitro* studies were used for *in vivo* evaluation. Following injections, a battery of behavioral tests including recording of ultrasonic vocalizations and neonatal developmental milestones in pups and evaluation of anxiety, exploration, grooming, social approach/novelty, depression like behavior, and motor strength in young rats were performed. Both male and female pups were studied, and the data were pooled. Corresponding to *in vitro* investigations, evaluation of neurodegeneration and oxidative stress was carried out in the brain tissue from young rats. The analyses were done in different batches of animals as described below.

**Animal housing and intracerebroventricular injections**. Normal Wistar rats were purchased from Charles River Laboratories (Germantown, MD, USA) and were bred at the New York State Institute for Basic Research Animal Colony according to the PHS Policy on Human Care and Use of Laboratory animals. Rats were housed (2/3 animals per cage) with a 12:12 h light/dark cycle and with ad libitum access to food and water. Studies on animals were carried out according to approved protocols from our Institutional Animal Care and Use Committee (IACUC) (Protocols no. 198 and 369).

On the day of birth, designated as P 0.5, pups were individually cryoanesthetized by placing them directly on wet ice for 3 min; the anesthetized pup’s head was placed on a non-heat conducting fiber optic light source and the lateral ventricles of the cerebrum were visualized by transillumination. A total of 4 μL of serum with or without P6 (concentrations described below) was injected unilaterally into the lateral ventricle through transcutaneous insertion with a specifically designed fine 10 μL Hamilton syringe with a 30-gauge/0.5inch/hypodermic cemented needle (Hamilton Syringe Co., Reno, NV, USA). For injections with sera without P6, 2 μL of autism or control serum was diluted with 2 μL of 0.9% NaCl (physiological saline) to make a final concentration of ~2% serum in rat pup CSF (assuming the total CSF volume in rat pup to be ~100 μL). For injections of sera with P6, 2 μL of autism or control serum was mixed with 2 μL of 1 μM P6 stock solution (final concentration of P6 in rat pup CSF~20 nM). For sham injection group, 4 μL of 0.9% NaCl was injected. From each litter, equal numbers of pups were injected for each group (sham, autism serum, control serum, autism serum+P6, control serum+P6) to diminish the litter effect among study groups. After intracerebroventricular injections (i.c.v.), pups were returned to the mother, and later behavioral tests were carried out. To avoid the potential confounding effects of previous handling, different badges of animals were used for ultrasound vocalizations and for neurobehavioral development and young rat behavior.

**General examination**. The physical state and condition of the rats were carefully examined throughout the study period by evaluating grooming, posture, physical state, and clasping reflex. Body weight was recorded daily during the initial 21 days (till weaning).

**Neurobehavioral development**. Examination of neurobehavioral development in rodents is an important study tool to model neurodevelopmental disorders like autism and Down’s syndrome which are characterized by growth retardation and delays in the appearance of developmental milestones [41,42]. In mice and rats, the early postnatal period is characterized by a spurt of brain growth, synaptogenesis, myelination, and the development of motor and sensory abilities [41,42]. Thus, evaluation of neurobehavioral development in rodents provides an opportunity to track the ontogeny of the nervous system through examination of neurological reflexes, early motor behavior including muscular strength and coordination, and developmental signs [42].

Evaluation of neurobehavioral development was performed following the procedure described in the relevant literature [42–51]. Examination was started on postnatal day 1 and was carried out until postnatal day 17 (or until the appearance of developmental milestone/reflex) daily between 12:00–15:00 in a set up made specifically for the purpose in the behavior lab. Weight was also recorded each day. The rat pups were evaluated for the following neurological signs, reflexes, and developmental milestones.

Surface righting is a measure of labyrinthine and body righting mechanisms, motor strength and coordination [42]. Each rat pup was placed on its back with the experimenter’s fingers holding the head and the hind body. The pup was released gently and the time taken in seconds to turn over with all four paws placed on the surface of the table was measured. The test was stopped if the pup did not turn over within maximum 30 s. It was measured once daily until the rat pup could right itself in less than 1s for two consecutive days. The data for the day of first appearance of the reflex was analyzed.

Negative geotaxis measures labyrinthine reflex and body righting mechanisms, strength, and motor coordination [42]. Each rat pup was placed head down on a square of screen mounted at an angle of 45^0^. The time taken by the pup to turn around 180^0^ to the head up position was recorded. The test was stopped if the pup did not turn around within 30 s. If the rat pup lost grip and slipped on the screen, it was replaced at the start point once. The test was repeated daily until the rat pup could perform appropriately in less than 30 s for two consecutive days. The first day of appearance of the reflex was analyzed for different groups.

Cliff aversion test is a measure of labyrinthine reflex function, feel sensitivity, and motor strength and coordination [42,51]. The rat pup was placed on the edge of a cliff (smooth box) with the snout and fore limbs over the edge, and the time taken in seconds to turn and crawl away was recorded. The test was repeated daily until the rat pup could perform appropriately in less than 30 s for two consecutive days. The first day of appearance of the reflex was analyzed for different groups.

Rooting reflex is a sensory tactile reflex also requiring motor coordination; it is mediated by the trigeminal nerve (cranial nerve V) [42,51]. A cotton swab was applied from front to back along the side of the head and the head turning response towards this tactile stimulus was recorded. The test was continued daily till the pup responded correctly for 2 consecutive days.

Ear twitch reflex, a measure of sensory tactile reflex, was tested daily by gently brushing the pulled out end of cotton swab against the tip of the ear; a positive reflex consisted of rat pup flattening the ear against the side of the head [42]. The test was repeated daily until the pup responded correctly for 2 consecutive days.

Eye opening is a developmental milestone. The pups were inspected daily for first day of opening of both eyes.

Like surface righting, air righting is also a measure of labyrinthine and body righting mechanisms and motor coordination [42]. The rat pup was held upside nearly 12 cm above the soft bedding of a cage and was released; the test was considered positive on the day when the pups land with all its four paws placed on the surface of the bedding. The test was repeated daily until the pup responded correctly for 2 consecutive days.

Fore limb grasp, a measure of strength, was tested by holding a rat pup with its forepaws grasping a string fixed from one end to the other end of the cage nearly 12 cm above the bedding. The pup was released, and the amount of time the pup spent grasping the string was recorded. The test was considered positive when the pup kept on grasping the string with fore limb for >1 s; it was repeated every day until performed correctly for 2 consecutive days [42].

Fore limb placing is a measure of placing reflex development which measures sensory and motor coordination. Fore limb placing test was performed by touching the dorsum of the paw with the edge of the table with the animal suspended; the first day of raising the forepaw and placing on the surface of the table was noted [43,51]. The test was repeated daily until the pup responded correctly for 2 consecutive days.

Auditory startle, an auditory reflex, was evaluated by clapping within 10 cm of the rat pup and the first day of the startle response was recorded [42,43]. The test was repeated daily until the pup responded correctly for 2 consecutive days.

**Ultrasonic vocalizations**. Ultrasonic vocalizations (USVs) have been widely used for behavioral phenotyping of rodent models of neurodevelopmental disorders [52–56]. USVs emitted by infant rats have been reported to be a reliable index of emotional development and communicative behavior [56,57]. Infant rats emit USVs in many different situations including isolation from dam and littermates, physical manipulation, and thermal and olfactory challenges [55,56,58–61]. Isolation induced USVs are considered to be distress vocalizations and have been shown to elicit maternal searching and retrieval [55,56,58,59]. In rat pups, USVs in response to isolation distress are evident usually on the first postnatal day; they increase in number and intensity toward the beginning of second week of life and then abruptly disappear by the end of second week [62–64]. USVs produced by rat pups typically fall in the range 20–50 KHz [65,66].

We recorded USVs daily from 2^nd^ to 11^th^ postnatal day in experimental animals based on the baseline measurements obtained initially with 12 untreated Wistar rat pups during a 5 min session daily from postnatal day 1 to 15. On each day of testing, pups were isolated one-by-one from their home cage and placed into an empty rectangular glass container (length x width x height = 10 cm x 7.5 cm x 6 cm) located inside a sound-attenuating Styrofoam box mounted with a bat detector. Three such systems were used at the same time, thus, allowing recording of USVs emitted by 3 pups simultaneously. Each box was closed to prevent the detectors from detecting sounds that were not derived from the pup inside. The temperature of the room was fixed at 22±1°C. The frequency detectors were set to 40 KHz, which is within the vocalization range of isolation induced USVs produced by rat pups [65,66]. The frequency detectors were attached via a Noldus box to a computer equipped with Ultravox software, which detected the number and duration of USVs. Minimum USV duration (“on” time; shortest time of the noise to be counted as a call) was set to be 10ms. For a call to be considered independent, an “off” time (minimum time silent before a new noise is counted as a call) of 5ms was set to be required. No differences were observed in the patterns of calling between male and female pups; thus, the data was pooled together across gender.

**Elevated plus-maze**. The level of anxiety in 18–19 day old young rats was evaluated by elevated plus maze testing. The elevated plus maze comprised of four arms (30x5 cm) connected by a common 5x5 cm center area. There were two opposite facing open arms (OA) and the other two facing arms enclosed by 20 cm high walls (CA). The entire plus-maze was elevated on a pedestal to a height of 82 cm above floor level in a room separated from the experimenter. The anxiogenic feature of the light for rats was maintained by ambient luminosity at 60 Lux which is considered to be non-anxiogenic. The young rat was placed onto the central area facing an open arm and was allowed to explore the maze for a single 8 min session. Between each rat, the feces were removed from the maze and the maze floor was wiped with paper towel soaked with 70% ethanol to avoid any urine or scent cues. For each rat, the number of OA and CA entries and the amount of time spent in each arm were recorded by a video tracking system (ANY-Maze software, version 4.5, Stoelting Co., Wood Dale, IL, USA). The anxiety-like behavior was evaluated by calculating the percentage of time spent in OA [OA/(OA+CA) x100]; OAs are more anxiogenic for rodents than CAs.

**Open field**. Exploratory behavior was analyzed by allowing 19–20 day old young rats to freely explore an open field arena in a single 15 min session. The testing apparatus was a classic open field consisting of a 50x50 cm PVC square arena surrounded by 40cm high walls. The open field was placed in a room separated from the investigator and was surmounted by a video camera connected to a computer tracking animals using a video tracking system (ANY-Maze software, version 4.5, Stoelting Co., Wood Dale, IL, USA). The parameters analyzed included time spent in the center of the arena and total distance traveled which are measures of anxiety and exploratory activity, respectively.

**Grooming, social approach, and social novelty test**. Grooming, social approach, and social novelty were analyzed using a 3-chamber box in 21–23 day old young rats [67–72]. The rectangular testing box consisted of clear plastic divided into three adjacent chambers (each 20 cm long, 40 cm wide and 22 cm high) and connected by open doorways (7 cm wide and 6.4 cm high). Social approach behaviors were tested in a single 35 min session, divided into 4 phases. This experiment had two habituation phases (center and all 3 chambers) followed by two testing phases (sociability and novelty). The first test or social approach phase of the test compared the preference for a social stimulus versus an inanimate object. The second test or social novelty phase of the test compared the preference for a now familiar social stimulus to a novel social stimulus.

The subject young rat was acclimated to the apparatus for 5 min in the center chamber (phase 1), and then for an additional 10 min with access to all 3 empty chambers (phase 2). The subject was then confined to the middle chamber, while the novel object (an inverted wire cup, Galaxy Cup, Kitchen Plus, Streetsboro, OH) was placed into one of the side chambers, and the stranger mouse (stranger 1), inside an identical inverted wire cup, was placed in the opposite side chamber. Age and gender matched Wistar rats were used as the stranger rat. The location (left or right) of the novel object and stranger rat alternated across subjects. The chamber doors were opened simultaneously, and the subject had access to all 3 chambers for 10 min (phase 3). After this, the fourth 10-min session provided a measure of preference for social novelty (phase 4). The subject rat was gently guided to the center chamber, the doors closed, and the novel object removed, and a second novel rat (stranger 2) was placed in the side chamber. The chamber doors were opened simultaneously, and the subject again had access to all 3 chambers for 10 min. The fourth 10 min phase provided a measure of recognition and discrimination. Video tracking with ANYmaze (Stoelting, Inc.; Wood Dale, IL) automatically scored the time spent in each of the 3 chambers, frequency and duration of grooming episodes; frequency and duration of sniffing episodes; and number of entries into each chamber during each phase of the test. Animals used as strangers were age and gender matched rats habituated to the testing chamber for 30 min sessions on 3 consecutive days and were enclosed in the wire cup to ensure that all social approach was initiated by the subject rat. An upright plastic drinking cup weighed down with a lead weight was placed on top of each of the inverted wire cups to prevent the subject rat from climbing on top. Ambient luminosity was maintained at 60 Lux.

**Forced swim test (behavioral despair test)**. Depression-like behavior was analyzed using behavioral despair test (Forced swimming test, FST, or Porsolt test) in 24–25 day old young rats. The FST, as originally described by Porsolt *et al* [73,74], assesses the tendency to give up attempting to escape from an unpleasant environment, whereby fewer attempts are interpreted as behavioral despair. The test was conducted in a single 6-minute session. Briefly, test rats were transported to a separate treatment room at least 1 hour before testing. The rat was placed in a cylinder of water (23 cm high, 11.5 cm diameter) filled to a depth of 16 cm that was meticulously maintained at 24±1°C. The time mice spent floating on the water (immobility time, sec) during 6 minutes as well as latency (sec) to the first immobility episode were manually observed by the investigator. After the testing, the animal was dried briefly with a towel and returned to its home cage. As is standard in the literature, all rats were exposed to the forced swim stressor in a cylinder that had been freshly cleaned and disinfected prior to the session. As described by Porsolt *et al* [73,74], an animal was considered immobile when floating motionless or making only those movements necessary to keep its head above the water surface. Swimming was defined as vigorous movements with forepaws breaking the surface of the water. Finally, the data was analyzed for immobility time (sec) for the last 4min of the 6 min testing session.

**Prehensile traction test**. Prehensile traction force was evaluated measuring fall latency of the 24–25 day old young rats suspended with forepaws from a string suspended 60 cm from a padded surface. The latency for the rat to fall from the string was measured up to 60 s.

**Tissue processing**. After completion of behavioral testing, the 26–27 day old young rats were perfused and brain tissue was collected for immunohistochemical and biochemical analysis. Animals were anesthetized with an overdose of sodium pentobarbital (125 mg/kg) and transcardially perfused with 0.1 M phosphate buffered saline (PBS). After perfusion, the brains were removed from the skull immediately. The left hemisphere was dissected into hippocampus, cerebral cortex, cerebellum, and brain stem, immediately frozen on dry ice, and then stored in -80°C ultrafreezer till used for biochemical analysis. The complete right hemisphere was immersion fixed in 4% paraformaldehyde in 0.1 M PBS for 24–48 hours, followed by cryoprotection in a 30% sucrose solution at 4°C overnight. Later, the 40 μm thick sagittal sections were cut on a freezing microtome. The sections were stored in glycol anti-freeze solution (Ethylene glycol, glycerol, and 0.1 M PBS in 3:3:4 ratio) at -20°C until further processing for immunohistochemical staining.

**Quantitative real time polymerase chain reaction (RT-qPCR) analysis of rat brain tissue**. The total RNA was extracted from a small piece of left cerebral cortex using RNeasy plus mini kit (Qiagen, Valencia, CA, USA) according to manufacturer’s instructions. Complementary DNA synthesis was carried out employing SuperScript first strand kit (Invitrogen, Carlsbad, CA, USA). The RT-*q*PCR was done using Brilliant SYBR Green Master Mix (Agilent, Santa Clara, CA, USA) in a Stratagene Mc3000p PCR detection system under the following conditions: 10 min at 95°C, 40 cycles of denaturation at 95°C for 30 s, annealing 55°C for 1 min, extension at 72°C for 1 min. The primer sequences were the following: forward 5’- GCGGCAGATAAAAAGACTGC-3’ and reverse 5’-GCCAGCCAATTCTCTTTTTG-3’ for BDNF; forward 5’-GGGACAGTTGATTTAGGGG-3’ and reverse 5’- GGCAGAAACTTGAGCATA-3’ for CNTF; forward 5’-GACATGCCGCCTGGAGAAC-3’ and reverse 5’-AGCCCAGGATGCCCTTTAGT-3’ for glyceraldehyde 3-phosphate dehydrogenase (GAPDH). Relative quantification was performed using the ΔΔCt method.

**Western blot analysis of rat brain tissue**. The remaining tissue from left cerebral cortex from each rat was homogenized in a Teflon-glass homogenizer to make 10% (w/v) homogenate. The pre-chilled homogenization buffer contained 50 mM Tris—HCl (pH 7.4), 8.5% sucrose, 2 mM EDTA, 2 mM EGTA, 10 mM b-mercaptoethanol plus the following protease and phosphatase inhibitors: 0.5 mM AEBSF, 10 μg/ml aprotinin, 10 μg/ml leupeptin, 4 μg/ml pepstatin, 5 mM benzamidine, 20 mM beta-glycerophosphate, 50 mM sodium fluoride, 1 mM sodium orthovanadate, and 100 nM okadaic acid. Protein concentration of each brain homogenate was estimatedby modified Lowry assay [75]. The tissue homogenates were boiled in Laemmli’s buffer for 5 min, and then subjected to 12.5% SDS-polyacrylamide gel electrophoresis (PAGE), followed by transfer of separated proteins on 0.45 μm Immobilon-P membrane (Millipore, Bedford, MA, USA). The following primary antibodies were used: rabbit polyclonal anti- BDNF, N-20 (1:1000, Santa Cruz Biotechnology, Santa Cruz, CA, USA); rabbit polyclonal anti-CNTF, FL-200 (1:500, Santa Cruz Biotechnology, Santa Cruz, CA, USA); mouse monoclonal anti-GFAP (1:5000, Millipore, Temecula, CA, USA); rabbit polyclonal anti-Iba1 (1:500, Wako Chemicals, Richmond, VA, USA); and rabbit polyclonal antibody to GAPDH (1:1000, Santa Cruz Biotechnology, Santa Cruz, CA, USA) as loading control. The blots were developed and quantified as described before. For quantification of different protein levels, each immunoreactive band was normalized to it's corresponding GAPDH band.

**Fluoro-Jade C labeling for neurodegeneration in rat brain tissue**. Fluoro-Jade C staining was performed as described previously [76,77] on 4–5 sections/animal and minimum of 6 animals/group (including 2 animals for each serum sample injected). Briefly, free floating brain sections were washed in large volumes of distilled water, followed by 3min incubation in 100% alcohol, 1 min in 70% alcohol, 1 min in 30% alcohol, and a 1 min wash in distilled water. The tissue sections were then incubated in 0.06% potassium permanganate solution for 15mins with gentle shaking followed by 1min wash in distilled water. The staining solution contained a 0.001% Fluoro-Jade C (Chemicon Millipore, Temecula, California) in 0.1% acetic acid. After 30 min incubation in Fluoro-Jade C solution with gentle shaking, the sections were washed three times for 1 min in distilled water followed by three 2 min rinses in xylene. The sections were mounted and cover slipped using DPX Fluka (Milwaukee, WI). Maximum projection images were generated based on confocal z-stacks using Nikon 90i fluorescent microscope equipped with Nikon C1 three-laser confocal system and a Nikon DS U1 digital camera. Images were filtered with a predetermined threshold using NIH Image J (v.1.46r) to create a binary image identifying positive and negative labeling and percentage of area occupied by Fluoro-Jade C positive labeling was calculated. Mean positive label values were averaged from 3–4 non-overlapping representative fields (40X objective) from cerebral cortex. The data from serum±Peptide 6 treatment groups was calculated as percentage of sham treated group.

**Measurement of oxidative stress using 8-OHdG in young adult rat brain tissue**. Immunohistochemistry for the DNA oxidative damage marker, 8-hydroxy-2^’^-deoxyguanosine (8-OHdG) was performed on free-floating sections and every tenth brain section was chosen for quantification. For quantification, 5–6 brain sections of minimum 6 animals per group (including 2 animals for each serum) were analyzed. The mouse monoclonal 8-OhdG primary antibody (1:500, QED Biosceince Inc., San Diego, CA) and Alexa 488-conjugated goat anti-mouse IgG secondary antibody (1:500, Molecular Probes, Carlsbad, CA, USA) were used. For quantification of 8-OHdG positive cells in cerebral cortex, four non-overlapping representative fields were imaged using 40X objective and maximal projection images were generated as described before. The number of positive cells in each cerebral cortex high power field (hpf) were counted and were averaged.

### Statistical analysis

Statistical analyses were performed using GraphPad Prism version 5.0 (GraphPad software inc., La Jolla, CA, USA) and SPSS version 17.0 (© SPSS Inc., 1989–2007, Chicago, Illinois, USA). Data are presented as mean±S.E.M. The normality of the data was determined using Kolmogorov-Smirnov test. The analysis involving multiple groups was done using one-way or two-way ANOVA followed by Bonferroni’s post-hoc test. Student’s *t*-test was used for all other comparisons (including inter-group comparisons for sera/peptide treatment effect). The statistically significant outliers excluded from the analysis were identified using Grubb’s test. For all purposes, *p* < 0.05 was considered as statistically significant.

## Results

### Sera from autistic children induce cell death and oxidative stress which can be rescued by P6 pre-treatment in mouse primary cultured cortical neurons

**Fig 1:**
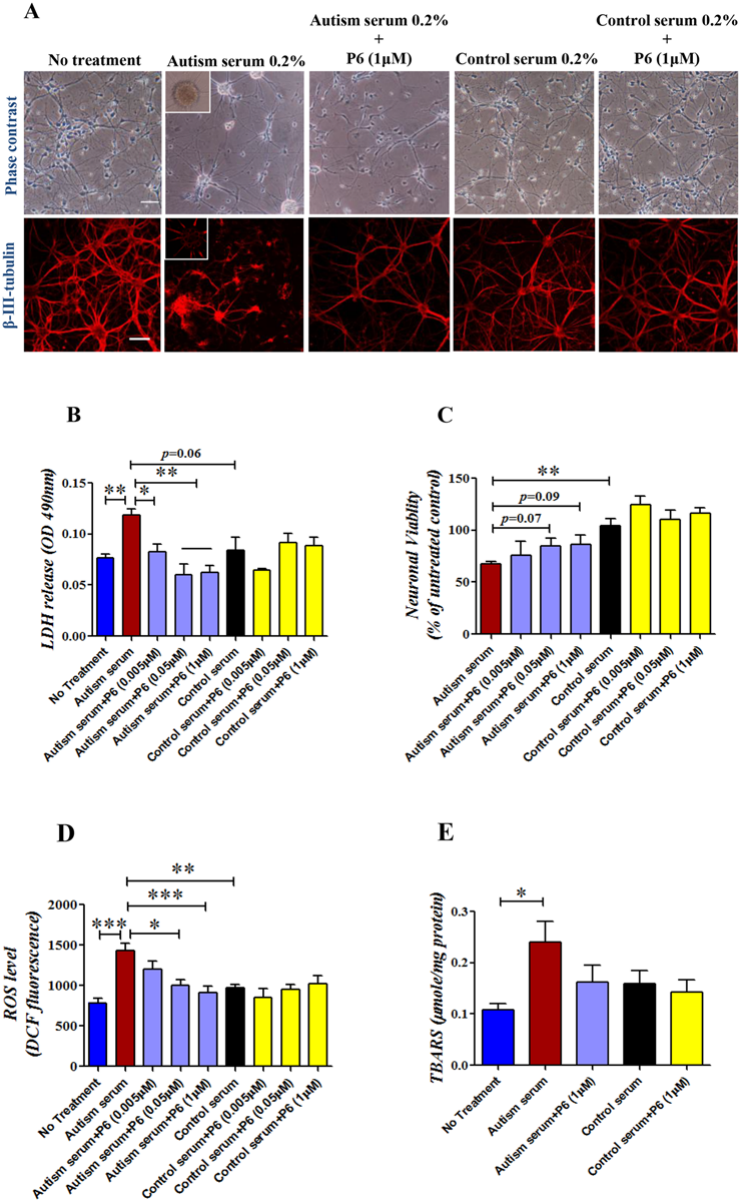
Effect of autism and control sera treatment with or without P6 on neuronal death/viability and oxidative stress in mouse primary cultured cortical neurons. (**A**) Representative images of phase contrast microscopy and β-III-tubulin (mature neuronal marker) staining of DIV7 primary cultured cortical neurons treated with 0.2% sera from autistic or control children with or without 1 μM P6 for 72 hours. Data is based on evaluation of the effect of 22 pairs of autism/control sera in 3 independent set of experiments. Autism sera markedly reduced the length of the neuritis and the number of cells and showed increased number of cell spheres and P6 could rescue these changes (**B and C**) Quantification of LDH cytotoxicity assay for evaluation of cell death (LDH release) and neuronal viability in DIV7 primary cultured cortical neurons treated with 0.2% sera from autistic or control children with or without 1 μM P6 for 72 hours. Data are shown as mean±S.E.M. based on the effect of 3 pairs of autism/control sera in 3 independent sets of experiments. (**D and E**) Data for DCF-DA assay for free radical production and TBARS assay for lipid peroxidation 3 days after treatment (DIV7) with sera from autistic or control children (0.2%) with or without P6 pre-treatment (0.005 μM, 0.05 μM, and 1 μM) is shown. Data are shown as mean±S.E.M. based on two independent sets of experiments evaluating 3 pairs of autism/control sera. *p<0.05, **p<0.01, and ***p<0.001. ANOVA with Bonferroni’s post-hoc test and/or Student’s *t*-test. Scale bar = 100 μm.

Previously, sera from individuals with autism which possess abnormal levels of various regulatory elements were shown to alter the development and proliferation of human neural progenitor cells (NPCs) and to possess autoantibodies against human NPCs [78–80]. In the present study, we observed that mouse primary cultured cortical neurons grown for 72 hours in medium supplemented with sera from autistic children either formed neurospheres like colonies of cells with sharp spinous processes or multiple small cells with markedly short processes and decreased cell density as compared to the untreated or control sera treated cell cultures both as observed by phase contrast microscopy and by immunostaining for neuronal marker, β-III-tubulin (Fig. 1A). Primary cultured neurons grown in the presence of sera from normal healthy controls revealed no gross morphological changes and only a few neurosphere like colonies were observed. Pretreatment with 1 μM P6 for 3 hours prevented the decrease in neurite length and cell density caused by the autism sera (Fig. 1A). These results were confirmed with 22 pairs of sera (autism and age-matched control, Table 1) in 3 different sets of primary cultures. Further analyses of cell death and oxidative stress were performed in 3 pairs of sera (Table 2) which showed the most marked consistent effect on neuronal morphology. A significant increase in cell death was found by LDH cytotoxicity assay in cultured neurons grown in the presence of sera from autistic children compared to untreated neurons (Fig. 1B; Bonferroni’s post-hoc test, *p*<0.05; Student’s t-test, *p* = 0.0039). Pretreatment with different doses of P6 resulted in a significant reduction in cell death in cultured neurons treated with sera from autistic children (Fig. 1B; P6 0.005 μM, Bonferroni’s post-hoc test, *p*>0.05, Student’s *t*-test, *p* = 0.0183; P6 0.05 μM, Bonferroni’s post-hoc test, *p*<0.01; P6 1 μM, Bonferroni’s post-hoc test, *p*<0.01). The cell death was not significantly altered in cultured neurons treated with sera from normal healthy controls compared to untreated controls (Fig. 1B; Bonferroni’s post-hoc test, *p*>0.05). Also, the cell death was less in control sera treated neurons compared to neurons treated with sera from autistic children (Fig. 1B; Student’s t-test, *p* = 0.0642, marginal significance). The neuronal viability, measured as a percentage of viability in untreated cells, was also significantly decreased in autistic sera treated neurons compared to those treated with control sera (Fig. 1C; Bonferroni’s post hoc test, *p*<0.01, Student’s t-test, *p* = 0.0076). P6 pretreatment showed improvement in neuronal viability in autism sera treated cultured neurons (Fig. 1C; P6 0.005 μM, Bonferroni’s post-hoc test, *p*>0.05, Student’s t-test, *p* = 0.5619; P6 0.05 μM, Bonferroni’s post-hoc test, *p*>0.05, Student’s t-test, *p*<0.0765; P6 1 μM, Student’s t-test, *p*<0.0964). Thus, we found that primary cultured neurons grown in the presence of sera from autistic children showed neuronal loss which was rescued by pretreatment with P6.

Primary cortical neurons grown in the presence of autistic sera showed higher levels of oxidative stress as analyzed by DCF-DA assay for free-radical production and TBARS assay for lipid peroxidation both compared to untreated neurons (Fig. 1D, DCF-DA, Bonferroni’s post-hoc test, *p*<0.001; Fig. 1e, TBARS, Bonferroni’s post-hoc test, *p*<0.05) and to neurons treated with control sera (Fig. 1D, DCF-DA, Bonferroni’s post-hoc test, *p*<0.001; Fig. 1e, TBARS, Bonferroni’s post-hoc test, *p*>0.05). Pretreatment with P6 resulted in a significant reduction in generation of free-radicals in autistic sera treated neurons (Fig. 1D; P6 0.05 μM, Bonferroni’s post-hoc test, *p*<0.05; P6 1 μM, Bonferroni’s post-hoc test, *p*<0.001). Even though a trend towards reduction in lipid peroxidation was noted in P6 pretreated autism sera treated neurons but it did not reach statistical significance (Fig. 1E; P6 1 μM, Bonferroni’s post-hoc test, *p*>0.05). Thus, it appeared that sera from autistic children could cause an increase in oxidative stress in primary cortical neurons which was counteracted by pretreatment with P6.

### Levels of neurotrophic factors are altered in sera from autistic children

**Fig 2:**
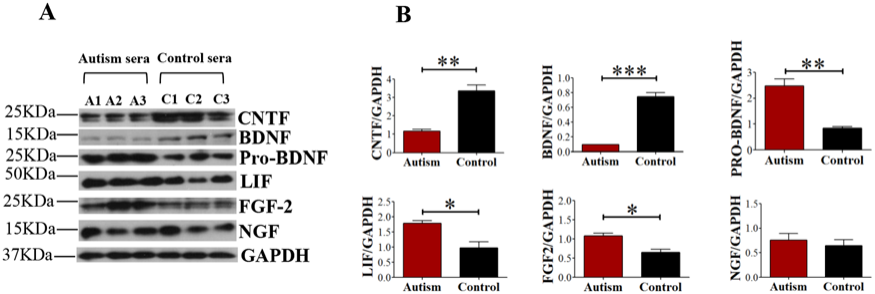
Levels of various neurotrophic factors in sera from autistic and control children. (**A and B**) Representative images and quantification of Western blots for evaluation of levels of various neurotrophic factors in 3 pairs of sera from autistic and control children used in the study are shown. A1, A2, A3, and C1, C2, C3, represent 3 autism and 3 control cases, respectively. *p<0.05, **p<0.01, and ***p<0.001. Student’s *t*-test.

The inappropriate brain milieu because of altered levels of various neurotrophic factors in the sera has been hypothesized to play a major role in abnormal brain development in autistic individuals. We thus evaluated the levels of various key neurotrophic factors in the 3 pairs of autism/control sera (Table 2) which induced increased cell death and oxidative stress in primary cultured cortical neurons. Indeed, the levels of various neurotrophic factors were found to be altered in sera from autistic children compared to those from age and gender matched control children as evaluated by quantitative Western blots (Fig. 2). The levels of mature CNTF and BDNF were markedly decreased in autism sera (Fig. 2A and B; CNTF, Student’s *t*-test, *p* = 0.0026; BDNF, Student’s *t*-test, *p* = 0.0003). Conversely, the levels of pro-BDNF, FGF-2, and LIF were found to be increased in autism sera compared to control (Fig. 2A and B; pro-BDNF, Student’s *t*-test, *p* = 0.0043; LIF, Student’s *t*-test, *p* = 0.0216; FGF-2, Student’s *t*-test, *p* = 0.0194). No statistically significant differences were observed in the levels of NGF (Fig. 2A and B; Student’s *t*-test, *p* = 0.5693). These data suggested the presence of neurotrophic abnormalities in the sera from autistic children that could have contributed to altered development of neurons and increase in cell death and oxidative stress found above (Fig. 1).

### Sera from autistic children induce developmental delay in rat pups which can be rescued by co-treatment with P6

**Fig 3:**
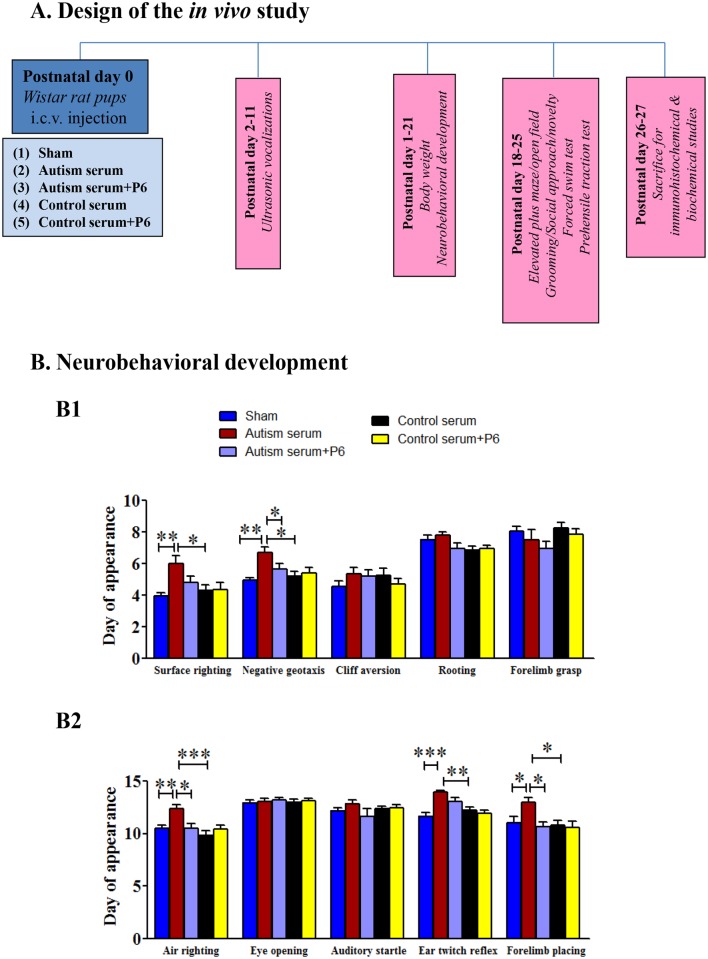
Design of the *in vivo* study and the effects of autism and control sera in the presence or absence of P6 on neurobehavioral development in rats. (**A**) Newly born Wistar rat pups were injected intracerebroventricularly on on postnatal day (P) 0.5 with saline (sham) or 2% autism or control serum with or without 20 nM P6. Behavioral studies were performed from postnatal day 2 to 25 in rats. (**B**) Evaluation of neurobehavioral development in Wistar rat pups from postnatal day 1–21(**B1**) Day of appearance of surface righting, negative geotaxis, cliff aversion, rooting, and forelimb grasp and (**B2**) air righting, eye opening, auditory startle, ear twitch, and fore limb placing. Data are presented as mean±S.E.M. based on sham (n = 17), autism serum (n = 15–16), autism serum+P6 (n = 16–17), control serum (n = 15–16), and control serum+P6 (n = 16–17). *p<0.05, **p<0.01, and ***p<0.001. ANOVA with Bonferroni’s post-hoc test and/or Student’s *t*-test.

The Wistar rat pups were injected intracerebroventricularly with autism or control sera with or without P6 within 24 hours of birth to evaluate their possible neurotoxic effect and potential neuroprotection by P6 *in vivo*. The *in vivo* studies were carried out using the same 3 pairs of autism/control sera as above for *in vitro* studies. Five groups of animals of 5–6/group were employed (Fig. 3A): (1) sham group injected with saline; (2) pups injected with sera from autistic children (5–6 rat pups for each serum); (3) pups injected with sera from normal healthy controls (5–6 rat pups for each serum); (4) pups injected with sera from autistic children plus P6 (5–6 rat pups for each serum); and (5) pups injected with sera from controls plus P6 (5–6 rat pups for each serum). The body weight recorded daily for infant rats (both male and female) from postnatal day 1 to postnatal day 21 did not differ significantly among the groups [S1 Fig; repeated measures 2-way ANOVA; group effect, *F* = 0.36 (4, 672), *p* = 0.836].

In neurobehavioral development study, autism serum injected pups displayed a significant delayed development of surface righting reflex compared to saline injected sham group and control serum group (Fig. 3B, panel 1; ANOVA, *p* = 0.0049, sham vs. autism serum group, Bonferroni’s post-hoc test, *p*<0.01, autism serum vs. control serum group, *p*<0.05). Autism serum with P6 injected group showed a trend towards earlier development of surface righting compared to autism serum alone group; however, the difference did not reach statistical significance (Fig. 3B, panel 1; autism serum vs. autism serum+P6, Bonferroni’s post-hoc test, *p*>0.05, Student’s *t*-test, *p* = 0.074).

Similarly, autism serum injected pups showed delayed appearance of negative geotaxis as compared to sham and control serum pups (Fig. 3B, panel 1; ANOVA, *p* = 0.0018, sham vs. autism group, Bonferroni’s post-hoc test, *p*<0.01, autism serum vs. control serum group, *p*<0.01). Autism serum with P6 group showed earlier development of negative geotaxis reflex compared to autism serum group (Fig. 3B, panel 1; Bonferroni’s post-hoc test, *p*>0.05, Student’s *t*-test, *p* = 0.044). Besides delay in appearance of negative geotaxis, autism serum group also took longer times to turn 180^0^ to head up position and move towards the top of the metallic grid in negative geotaxis testing over the period of development [S2A Fig; repeated measures 2-way ANOVA; group effect, *F* = 4.78 (4, 219), *p* = 0.001; postnatal day 8, sham vs. autism serum group, Bonferroni’s post-hoc test, *p*<0.05; postnatal day 14, sham vs. autism serum group, Bonferroni’s post-hoc test, *p*<0.01]. Autism serum with P6 group showed a trend towards better performance compared to autism serum alone group, however, it was not statistically significant.

The appearance of cliff aversion reflex did not differ significantly across groups (Fig. 3B, panel 1; ANOVA, *p* = 0.8410). The autism serum injected pups took longer time to show cliff aversion compared to sham group during the observed period of development; however, it was not statistically significant (S2B Fig; repeated measures 2-way ANOVA, group effect, F = 1.68 (4, 219), *p* = 0.1567; sham vs. autism serum group, Bonferroni’s post-hoc test, *p*>0.05), and P6 treatment had no detectable effect on cliff aversion reflex.

The tests for rooting reflex and forelimb grasp did not reveal any significant differences among groups (Fig. 3B, panel 1; rooting, ANOVA, *p* = 0.1044; forelimb grasp, ANOVA, *p* = 0.2626). Similarly, the appearance of eye opening and auditory startle did not differ between groups (Fig. 3B, panel 2, eye opening, ANOVA, *p* = 0.9708; auditory startle, ANOVA, *p* = 0.3677).

The development of air righting which like surface righting is a measure of labyrinthine reflex and motor coordination was significantly delayed in autism serum injected pups compared to sham group and control serum group; this developmental delay was significantly corrected by P6 treatment (Fig. 3B, panel 2; ANOVA, *p* = 0.0002; sham vs. autism group, Bonferroni’s post-hoc test, *p*<0.01; autism serum vs. control serum group, *p*<0.001; autism serum vs. autism serum+P6 group, Bonferroni’s post-hoc test, *p*<0.05).

Similarly, development of ear twitch reflex was markedly delayed in autism serum injected pups compared to sham and control serum groups but P6 had no significant effect (Fig. 3B, panel 2; ANOVA, *p*<0.0001; sham vs. autism group, Bonferroni’s post-hoc test, *p*<0.001; autism serum vs. control serum group, *p*<0.01; autism serum vs. autism serum+P6 group, Bonferroni’s post-hoc test, *p*>0.05).

Finally, fore limb placing was significantly delayed in autism serum injected animals compared to sham and control serum groups and the performance in autism serum with P6 group was improved (Fig. 3B, panel 2; ANOVA, *p* = 0.0075; sham vs. autism group, Bonferroni’s post-hoc test, *p*>0.05, Student’s *t*-test, *p* = 0.0143; autism serum vs. control serum group, *p*<0.05; autism serum vs. autism serum+P6 group, Bonferroni’s post-hoc test, *p*<0.05).

Overall, the developmental milestones in rats which were affected by treatment with autism serum involved complex motor performance and skills; on the contrary, most of the neurodevelopmental behaviors which were unaltered in autism serum injected pups are known to be mediated by simplex reflex circuitry [42,44,46,50,81]. P6 co-injected with autism serum was able to ameliorate the deficits in negative geotaxis, air righting and fore limb placement induced by autism serum.

### Sera from autistic children induce deficits in isolation-induced ultrasonic vocalization calls in rat pups

Social communication deficit is one of the fundamental clinical phenotype of ASDs [82,83].

**Fig 4:**
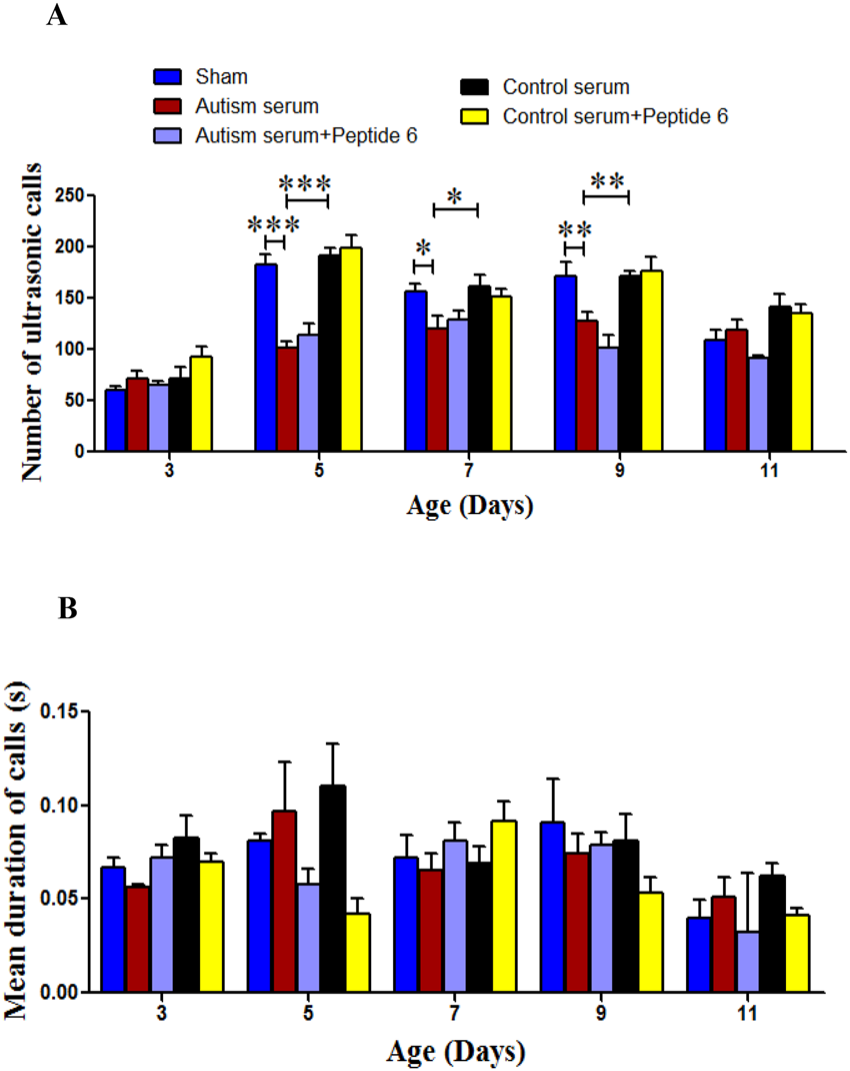
Effects of autism and control sera in the presence or absence of P6 on ultrasonic vocalizations (USVs) from postnatal day 2–11 in rat pups. (**A and B**) Social communication in young Wistar rats injected intracerebroventricularly on P 0.5 with sham or 2% autism or control serum with or without 20 nM P6. Social communication was evaluated by the number (**A**) and duration (**B**) of isolation induced ultrasonic calls emitted by rat pups during the 5min test on postnatal days 3, 5, 7, 9, and 11. Data are presented as mean±S.E.M. in saline (sham) (n = 15–17), autism serum (n = 15–17), autism serum+P6 (n = 15–17), control serum (n = 15–17), and control serum+P6 (n = 15–17) treated pups. *p<0.05, **p<0.01, and ***p<0.001. ANOVA with Bonferroni’s post-hoc test and/or Student’s *t*-test.

Although rodents such as rats and mice do not use language, they emit auditory signals including USVs [71,72]. USVs emitted by rat pups upon separation from the dam and littermates can be used to assess the ability of social communication [71,72]. We found that number of isolation-induced ultrasonic calls was significantly lower in pups injected with sera from autistic children compared to saline injected sham group and control sera injected group on postnatal days 5, 7, and 9 [Fig. 4A; repeated measures 2-way ANOVA, group effect, F = 28.84 (4, 365), *p*<0.0001; postnatal day 5, sham vs. autism serum group, Bonferroni’s post-hoc test, *p*<0.001, autism serum vs control serum group, Bonferroni’s post-hoc test, *p*<0.001; postnatal day 7, sham vs. autism serum group, Bonferroni’s post-hoc test, *p*<0.05, autism serum vs control serum group, post-hoc test, *p*<0.05; postnatal day 9, sham vs. autism serum group, Bonferroni’s post-hoc test, *p*<0.01, autism serum vs control serum group, post-hoc test, *p*<0.01]. No significant effect of P6 treatment was found on USVs emitted by the rat pups. The mean duration of ultrasonic calls did not differ between groups [Fig. 4B; repeated measures 2-way ANOVA, group effect, F = 1.79 (4, 365), *p* = 0.1306]. The decreased number of ultrasonic calls emitted after maternal and littermate isolation in rat pups injected with sera from autistic children suggest decreased propensity towards their mothers as is also common in autistic infants.

### P6 treatment can rescue social approach and novelty impairments in autism sera treated young rats

**Fig 5:**
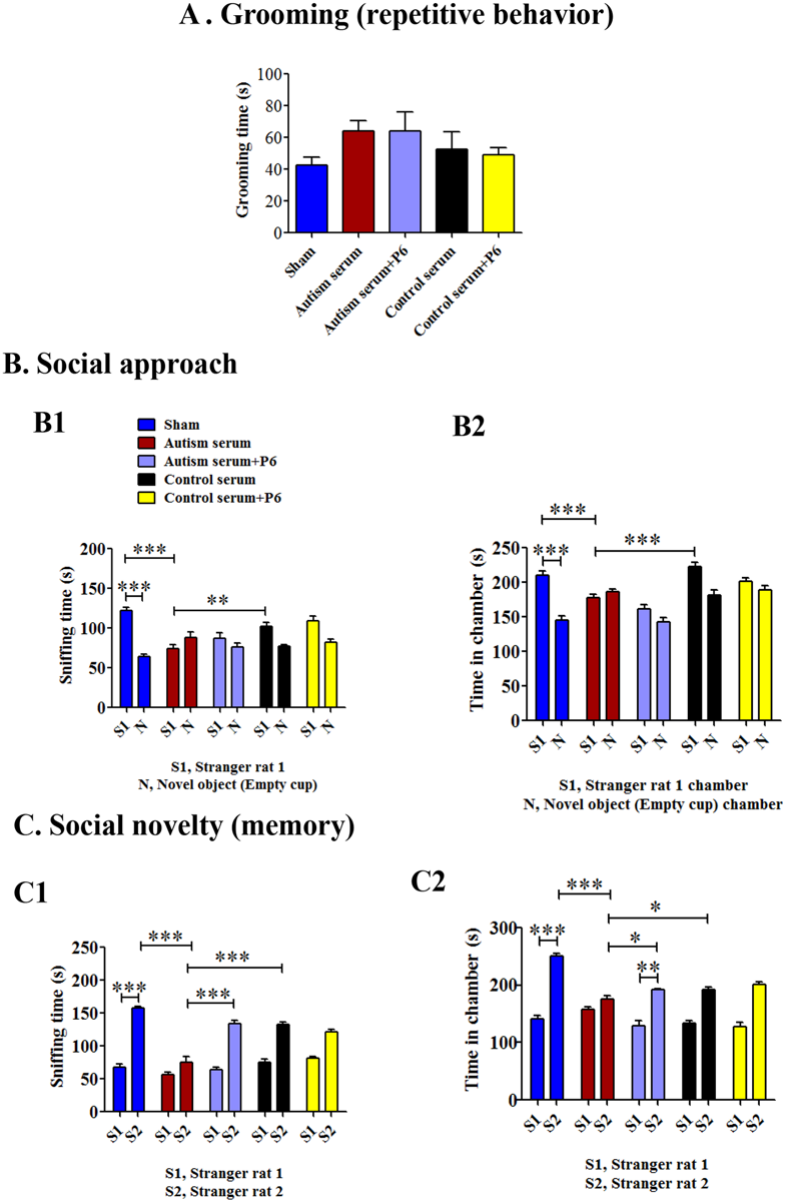
Effect of autism and control sera in the presence or absence of P6 on grooming, social approach and novelty in young Wistar rats. P 0.5 rat pups were injected intracerebroventricularly with saline (sham) or 2% autism or control serum with or without 20 nM P6. (**A**) Grooming time measured during the first 5 min habituation phase in the central chamber of the 3-chamber social approach/novelty task. (**B**) Sniffing time and time in the chamber (“stranger rat 1” versus “novel object”) spent in the social approach task. (**C**) Sniffing time and time in the chamber (“stranger rat 1” versus “stranger rat 2”) spent in the social novelty task. Data are presented as mean±S.E.M. based on sham (n = 15), autism serum (n = 15), autism serum+P6 (n = 16), control serum (n = 15), and control serum+P6 (n = 16). *p<0.05, **p<0.01, and ***p<0.001. ANOVA with Bonferroni’s post-hoc test and/or Student’s *t*-test.

During the first habituation phase of the 3-chamber social approach/novelty task, the grooming time as measured during the 5 min exploration of the central chamber did not differ between the different groups (Fig. 5A; ANOVA, *p* = 0.2988). Nonetheless, there was a strong trend towards increased grooming time in young rats injected with sera from autistic children compared to the sham group suggesting an increased tendency towards a spontaneous repetitive behavior (Fig. 5A; Bonferroni’s post hoc test, *p*>0.05; Student’s t-test, *p* = 0.014).

In the 3-chambered social arena test, young rats injected with sera from autistic children displayed dysfunctional social interaction behavior (one of the most recognizable manifestations of autistic behavior) compared to sham and control serum injected groups (Fig. 5B and C). The young rats injected with autistic sera spent much less time interacting with social partner (“stranger 1”) compared to sham and control serum groups; P6 treatment had no effect on this autistic behavior (Fig. 5B, panel1; sham vs. autism serum group, Bonferroni’s post-hoc test, *p*<0.001, autism serum vs control serum group, Bonferroni’s post-hoc test, *p*<0.001; autism serum vs autism serum+P6, Bonferroni’s post-hoc test, *p*>0.05). Similar trends were observed for time spent in social partner chamber and empty cup chamber (Fig. 5B, panel 2; sham vs. autism serum group, Bonferroni’s post-hoc test, *p*<0.001, autism serum vs control serum group, Bonferroni’s post-hoc test, *p*<0.001; autism serum vs autism serum+P6, Bonferroni’s post-hoc test, *p*>0.05).

In a subsequent trial, when a novel social partner (“stranger 2”) was introduced, autism sera injected rats displayed a marked lack of preference for social novelty compared to sham and control serum groups; P6 treatment was able to rescue this deficit (Fig. 5C; sniffing time, sham vs. autism serum group, Bonferroni’s post-hoc test, *p*<0.001, autism serum vs control serum group, Bonferroni’s post-hoc test, *p*<0.001; autism serum vs autism serum+P6, Bonferroni’s post-hoc test, *p*<0.001; time spent in stranger 2 chamber, sham vs. autism serum group, Bonferroni’s post-hoc test, *p*<0.001, autism serum vs control serum group, Bonferroni’s post-hoc test, *p*<0.05; autism serum vs autism serum+P6, Bonferroni’s post-hoc test, *p*<0.05). These data suggest that the dysfunction in social novelty which is also a measure of short-term social memory induced by sera from children with autism was rescued by P6.

The autism sera did not induce any significant changes in the level of anxiety, exploratory activity, motor performance, or depression in rats (S3 Fig).

### Sera from autistic children induce neurodegeneration and increase oxidative stress and neuroinflammation in young rats brains which is counteracted by P6 probably via increase in BDNF expression

**Fig 6:**
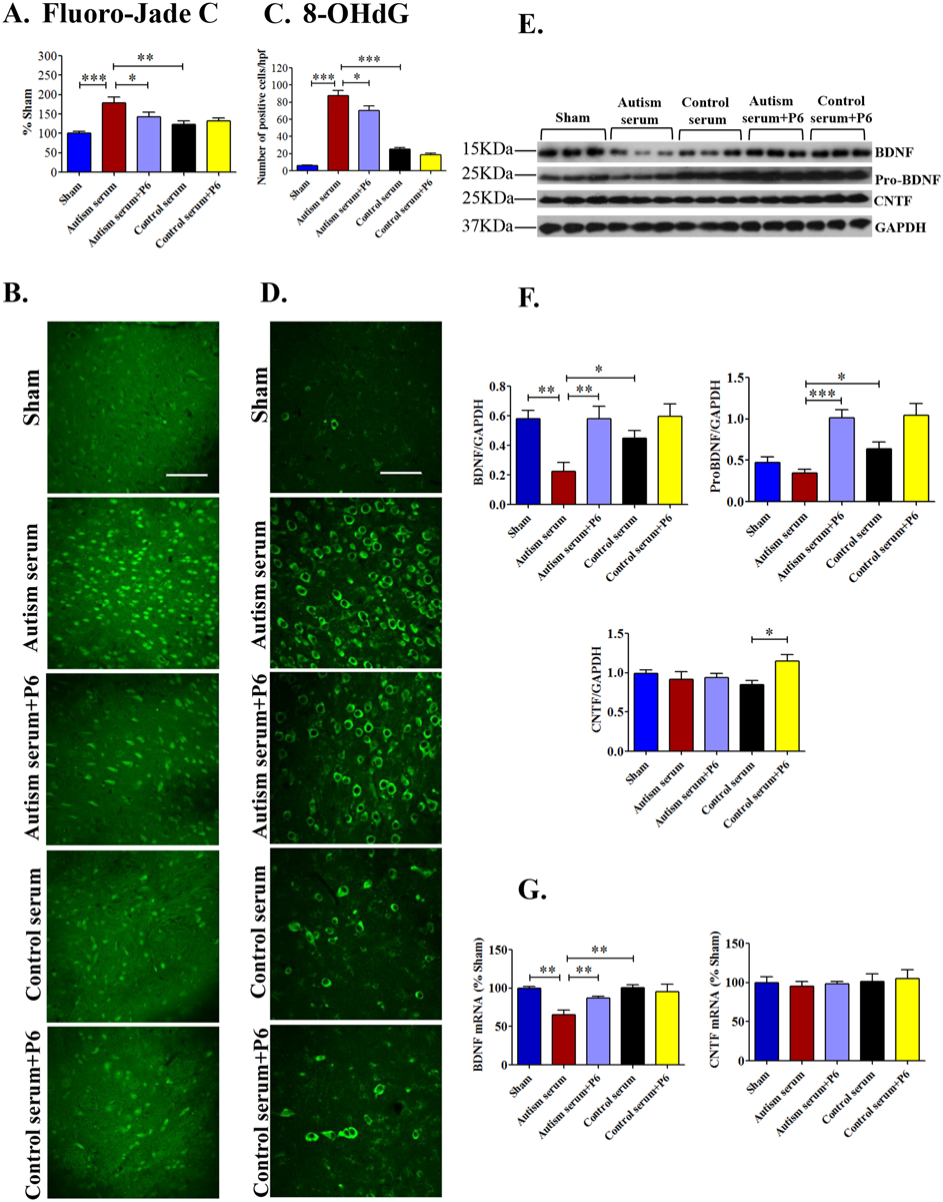
Effect of autism and control sera in the presence or absence of P6 on neurodegeneration, oxidative stress, and CNTF, BDNF, and pro-BDNF expression in the cerebral cortex of young Wistar rats. P 0.5 rats were injected intracerebroventricularly with saline (sham) or 2% autism or control serum with or without 20 nM P6. On postnatal day 26–27, rats were sacrificed and their brains were evaluated by quantitative immunohistochemistry and Western blots (**A and B**) Quantification and representative images of Fluorojade C staining, a sensitive marker of neurodegeneration, in the cerebral cortex are shown. Quantification is based on minimum of 6 animals/group (including 2 animals for each serum sample injected). (**C and D**) Quantification and representative images of 8-OHdG positive neurons, a marker of DNA damage caused by oxidative free radicals, in the cerebral cortex are shown. Quantification is based on minimum of 6 animals/group (including 2 animals for each serum sample injected). (**E and F**) Representative Western blots and densitometric quantification of BDNF, pro-BDNF, and CNTF expression normalized to GAPDH in the cerebral cortex of young Wistar rats. Data are presented as mean±S.E.M. based on sham (n = 7), autism serum (n = 7), autism serum+P6 (n = 8), control serum (n = 6), and control serum+P6 (n = 7). (**G**) Messenger RNA expression levels of BDNF and CNTF presented as percent of sham group. Quantification is based on 6 rats/groups. *p<0.05, **p<0.01, and ***p<0.001. ANOVA with Bonferroni’s post-hoc test and/or Student’s *t*-test. Scale bar = 100 μm.

In parallel with the *in-vitro* studies utilizing primary cultured cortical neurons, we investigated the *in vivo* effect of sera from autistic and control children and potential neuroprotective effect of P6 on neurodegeneration and oxidative stress in the cerebral cortex of young rats. Fluorojade C histochemical staining, a sensitive marker of neurodegeneration, confirmed a marked increase in neurodegeneration in autism serum injected rats compared to sham and control serum group; P6 was able to significantly reduce this autism serum induced neurodegeration (Fig. 6A and B; ANOVA, *p*<0.0001; sham vs. autism group, Bonferroni’s post-hoc test, *p*<0.001; autism serum vs. control serum group, Bonferroni’s post-hoc test, *p*<0.01; autism serum vs. autism serum+P6 group, Bonferroni’s post-hoc test, *p*<0.05). Similarly, a marked increase in 8-OHdG positive neurons, a marker of DNA damage caused by oxidative free radicals, was observed in autism serum injected rats; P6 also exerted a beneficial effect here (Fig. 6C and D; ANOVA, *p*<0.0001; sham vs. autism group, Bonferroni’s post-hoc test, *p*<0.001; autism serum vs. control serum group, Bonferroni’s post-hoc test, *p*<0.001; autism serum vs. autism serum+P6 group, Bonferroni’s post-hoc test, *p*<0.05). Collectively, these data provided the anatomical and physiological basis for the behavioral abnormalities observed in autism sera injected rats and the potential therapeutic beneficial effect of P6.

We further evaluated the effect of sera with or without P6 treatment on the protein and mRNA expression levels of BDNF and CNTF in the cerebral cortex of young rats. The densitometric quantification of Western blots developed with anti-BDNF and normalized to GAPDH revealed decreased levels of both pro-BDNF and mature BDNF in autistic sera treated rat brains compared to control sera treatment group (Fig. 6E and F; Bonferroni’s post-hoc test, *p*<0.05 for both pro-BDNF and BDNF). P6 (20 nM) co-treatment was able to correct the autistic sera induced reduction in both pro-BDNF and BDNF expressions (Fig. 6E and F; pro-BDNF, Bonferroni’s post-hoc test, *p*<0.001; BDNF, Bonferroni’s post-hoc test, *p*<0.01). The mRNA level of BDNF was significantly lower in autistic sera treated rat brains compared to sham and control sera treatment groups (Fig. 6G; ANOVA, *p* = 0.0006; sham vs. autism group, Bonferroni’s post-hoc test, *p*<0.01; autism serum vs. control serum group, Bonferroni’s post-hoc test, *p*<0.01). P6 treatment significantly enhanced BDNF mRNA levels (Fig. 6G; autism serum vs. autism serum+P6 group, Bonferroni’s post-hoc test, *p*>0.05, Student’s *t*-test, *p* = 0.0049). These data suggests that the beneficial effect of P6 on abnormalities in autism sera treated rats could be because of rescue of BDNF level.

The CNTF levels did not differ significantly between sham, autism serum, autism serum+P6, and control serum groups (Fig. 6E and F; ANOVA, *p* = 0.068, Bonferroni’s post-hoc test, *p*>0.05); however, there was a significant increase in CNTF expression in control serum+P6 group compared to control serum alone group (Fig. 6E and F; Bonferroni’s post-hoc test, *p*>0.05; Student’s *t*-test, *p* = 0.0155). Similarly, CNTF mRNA level did not differ between groups (Fig. 6G; ANOVA, *p* = 0.922; Bonferroni’s post-hoc test, *p*>0.05).

We further evaluated the effect of autism sera treatment on two markers of neuroinflammation i.e., glial fibrillary acidic protein, GFAP, a marker of astrocytes, and Iba1, a marker of microglia, in the cerebral cortex of young rats by Western blots. The levels of GFAP were marginally increased in autism serum group compared to sham animals (S4 Fig; GFAP, ANOVA, *p* = 0.0104, Bonferroni’s post-hoc test, *p*>0.05, Student’s *t*-test, *p* = 0.073) and significantly increased compared to control serum group (Bonferroni’s post-hoc test, *p*<0.05). P6 treatment significantly reduced GFAP levels (S4 Fig; autism serum vs autism serum+P6 group, Bonferroni’s post-hoc test, *p*>0.05, Student’s *t*-test, *p* = 0.049). There were no significant differences between groups in the expression levels of Iba1 (S4 Fig; Iba1, ANOVA, *p* = 0.3012, Bonferroni’s post-hoc test, *p*>0.05)

## Discussion

ASDs constitute a major healthcare problem with millions affected in U.S. and worldwide. The exact etiopathogenesis of the disorder is not yet known and there is no effective pharmacological treatment available for these patients. Among the many hypothesized etiological factors, early neurotrophic imbalance by virtue of providing improper brain milieu has been speculated to play a major role in the pathogenesis of autism. The present study shows that alterations in the levels of neurotrophic factors in the sera from autistic individuals could contribute to neurobehavioral phenotype of autism in rats. We found that a CNTF small peptide mimetic, P6, could rescue the ASD specific deficits in rats probably by inducing an increase in BDNF level. These data provide rationale for neurotrophic factors based serum/plasma screening assay for autism and a potential therapeutic strategy via modulation of neurotrophic support. Furthermore, the intracerebroventricular treatment of newborn rats with sera from children with autism provides a potential useful animal model of the disease.

Neurotrophic factors play essential roles in all stages of central nervous system development and maintenance; they critically influence the formation and elimination of neuronal connections [84]. Several studies suggest that aberrant cerebral connectivity and synaptic plasticity constitute essential features of the pathogenesis of autism (for review, [1–5]). Thus, neurotrophic factors which are essential mediators of neuronal and synaptic plasticity have been hypothesized to play a major role in the pathophysiology of autism. Altered brain, CSF, and serum levels of neurotrophic factors have been reported in patients with autism. For example, serum level of BDNF, which plays an essential role in brain development, neurogenesis and synaptogenesis, and synaptic plasticity, was shown to be decreased in children, adolescents, and adults with autism [22, 85–88]. Contrarily, few studies have reported increased BDNF serum levels in patients with autism (particularly during the early neonatal period), and an increase in BDNF levels during early developmental stages has been speculated to contribute to the pathophysiology of autism [89–93]. Our data which is based on ~5year old children suggest that BDNF levels are decreased in autism, and this may contribute to abnormalities in neurogenesis and synaptogenesis and synaptic plasticity in autism. Interestingly, we found that the levels of pro-BDNF, the precursor to mature BDNF, were increased in sera from children with autism. This finding which is in concordance to the report by Garcia *et al* [94] suggests the defective processing of pro-BDNF to BDNF in autism. Nonetheless, we found that the protein levels of both pro-BDNF and BDNF and mRNA level of BDNF were decreased in autism sera treated rat brain tissue. Besides BDNF, we also observed abnormalities in the serum levels of various other neurotrophic factors in children with autism. The serum levels of CNTF were found to be lower and the levels of FGF-2 and LIF were found to be higher in children with autism compared to age-matched healthy controls. Previously, increased oxidative stress which is widely implicated in the pathophysiology of autism was shown to block CNTF activity in neurons which is essential for neuronal survival and maintenance [19–21]. We previously showed that increase in FGF2 levels inhibits neuronal lineage determination and maturation during neurogenesis, and it can be counteracted by both CNTF and P6 [23]. Our previous studies also showed that LIF, a cytokine, which maintains totipotency of neural stem cells and inhibits neuronal lineage while favoring glial lineage formation [95–99] can be inhibited with P6 [31]. Our data suggest that the levels of various neurotrophic factors are altered in sera from children with autism and this imbalance along with the increased oxidative stress could be among the primary factors responsible for the altered development and neurodegeneration observed in both *in vivo* and *in vitro* models.

Oxidative stress resulting from excess generation of reactive oxygen species (ROS) has been implicated in the pathogenesis of autism [8,100]. Previously, lipid peroxidation markers were reported to be elevated in plasma from children with autism indicating that oxidative stress is increased in this disease [101]. Contrarily, the serum levels of major antioxidant proteins, transferrin (iron-binding protein) and ceruloplasmin (copper-binding protein), were found to be significantly decreased in children with autism compared to their non-autistic siblings [101]. We found that culturing the mouse primary cortical neurons in the presence of sera from autistic children result in increase in levels of ROS and lipid peroxidation. Similarly, increased oxidative stress-induced DNA damage was observed in brain tissue from rats exposed to sera from autistic children during the early period of development. These findings support the notion that altered brain environment contributes to increased oxidative stress in early developmental stages in autism. Increased oxidative stress has been suggested to lead to membrane lipid abnormalities, mitochondrial dysfunction, excitotoxicity, and immune dysfunction in autism, and may ultimately contribute to the behavioral phenotype of autism [8].

Inflammatory changes especially astroglial activation have been described in the brains of patients with autism [102–104] and may contribute to the pathogenic mechanisms involved in cortical and neuronal dysfunction [105]. Astrocytes and microglia play critical roles in the neurobiological processes of cortical organization, neuroaxonal guidance, and synaptic plasticity [105–107]. Increased GFAP level observed in the present study in autism sera treated rats could signify gliosis, reactive injury and impaired neuronal migration processes [105,108]. The rescue of astorgliosis by P6 treatment in the present study signifies the potential therapeutic usage of neurotrophic factor based strategy for ameliorating neuroinflammation in ASD.

The present study suggests that dysfunction of brain environment in autism can contribute to behavioral deficits. Exposing the cultured neurons and early postnatal brains to sera from autistic children resulted in neurodegeneration, increased oxidative stress, and behavioral impairments. It has been hypothesized that the abnormal behavioral phenotype of autism may result from structural and functional alterations in brain caused by abnormalities in brain development during embryonic period and early postnatal life [109–111]. Increased oxidative stress and imbalance of neurotrophic factors could be major contributing factors to pathophysiology of autism. During early brain development, neurotrophic factors provide an appropriate brain milieu necessary for all aspects of neural development including neuronal proliferation, differentiation, growth, and migration [112–115]. Similarly, neurogenesis is highly sensitive to oxidative stress induced damage; hippocampal neurogenesis is reported to be reduced after exposure to oxidative stress in vivo in an environment lacking antioxidant enzymes [116,117]. Thus, we can hypothesize that impaired neurotrophic balance and increased oxidative stress could alter the early brain development leading to autistic behavioral phenotype.

The beneficial effect we observed with P6 treatment further strengthens the idea that autism could be caused by an early imbalance of neurotrophic factors and increased oxidative stress. P6 pretreatment prevented cell death induced by autism sera in primary cultured cortical neurons. P6 is an 11-mer CNTF derived peptide which has been demonstrated to exert neurogenic and neurotrophic effects both *in vivo* and *in vitro* [31–35,118,119]. The CNTF/JAK/STAT pathway has been implicated in the development, survival, and maintenance of neurons and glia in central nervous system [21,120,121]. We had reported previously that P6 which corresponds to a biologically active region of human CNTF exerts its neurogenic and neurotrophic effect via modulation of JAK/STAT and LIF signaling pathways and increased expression of BDNF [31,35]. In the current study, we found that P6 was able to rescue autism serum-induced neurodegeneration and oxidative stress in cultured neurons and rat brains. The neuroprotective effect of P6 could have been because of increased BDNF expression we observed in P6 treated rat brains. We previously showed that P6 and its fragment peptide, Peptide 021 (P021) enhance BDNF mRNA and protein levels [35,38]; BDNF is known to exert protective effect against oxidative stress [122,123]. Recently, a relationship has been suggested between BDNF, sonic hedgehog (SHH), and oxidative stress in autism [22]. Wu et al [124,125] showed that BDNF induces up-regulation of SHH at both mRNA and protein levels, and the protective effect of BDNF in cortical neurons could be abolished by using SHH signaling inhibitor. Based on this, we can speculate that the protective effect of P6 against autism serum-induced neurodegenration and oxidative stress could have been mediated via BDNF.

One of the most remarkable findings of the current study is the development of several features of autism in young rats whose brains were exposed to sera from autistic children via i.c.v. injections. This single finding strongly suggests the important role brain environment plays during early development in the pathophysiology of autism. Early postnatal exposure of brain tissue to sera from autistic children which had abnormalities in neurotrophic factor levels led to developmental delay and social communication, interaction, and memory deficits in young rats. Several of these deficits such as developmental delay and social memory deficits were rescued by P6 treatment. Interestingly, the early postnatal exposure to autistic sera resulted in increased oxidative stress induced DNA damage and neurodegeneration in cortical tissue of young rats providing the structural correlate for behavioral abnormalities observed in these rats. Remarkably, P6 treatment was able to rescue these structural abnormalities probably via increased BDNF expression.

In summary, this study provides evidence regarding the neurotrophic abnormalities in autism and the potential role they play in the pathophysiology of the disease. We speculate that the brain milieu of autistic children is altered and favors increased oxidative stress and neurodegeneration. Ameliorating the neurotrophic imbalance during early stages of brain development can serve as a potential therapeutic approach for autism. P6 represents a new class of neurotrophic peptide mimetics that has potential therapeutic value for ASD and related conditions.

## Supporting Information

S1 Fig The body weight evaluation of young Wistar rats from postnatal day 3 to 21. Data are presented as mean±S.E.M. based on sham (n = 17), autism serum (n = 16), autism serum+P6 (n = 17), control serum (n = 16), and control serum+P6 (n = 17).
(TIF)

S2 Fig Performance in negative geotaxis testing on postnatal days 8, 11, and 14 (A) and cliff aversion on postnatal days 9, 12, and 15 (B). Data are presented as mean±S.E.M. based on sham (n = 17), autism serum (n = 15–16), autism serum+P6 (n = 16–17), control serum (n = 15–16), and control serum+P6 (n = 16–17). *p<0.05, **p<0.01, and ***p<0.001. ANOVA with Bonferroni’s post-hoc test and/or Student’s *t*-test.
(TIF)

S3 Fig General behavioral characterization in young Wistar rats injected intracerebroventricularly on P 0.5 with sham or 2% autism or control serum with or without 20 nM P6. Anxiety-like behaviors were evaluated by (**A**) percent time in the open arm, OA (ANOVA, *p* = 0.8551), and (**B**) number of entries to OA (ANOVA, *p* = 0.5295) in an elevated plus maze on postnatal day 18–19, and (**C**) time in the center (ANOVA, *p* = 0.9975) in an open field arena on postnatal day 19–20. There was a trend towards decreased number of OA entries in autism serum injected young rats (sham group vs autism serum group, Bonferroni’s post hoc test, *p*>0.05, Student’s t-test, *p* = 0.08). (**D**) Spontaneous locomotor and exploratory activities were assessed in open field [repeated measures 2-way ANOVA, group effect, F = 0.34 (8, 219), *p* = 0.9513]. (**E**) Motor strength was evaluated by latency to fall in prehensile traction test on postnatal day 24–25 (ANOVA, *p* = 0.9332). (**F**) Behavioral despair and depression-like behavior was analyzed by immobility time in forced swim test on postnatal day 24–25 (ANOVA, *p* = 0.9410). Data are presented as mean±S.E.M. based on sham (n = 15–17), autism serum (n = 15–17), autism serum+P6 (n = 15–17), control serum (n = 15–17), and control serum+P6 (n = 15–17).
(TIF)

S4 Fig Effect of treatment with autism and control sera with or without P6 on markers of neuroinflammation in the cerebral cortex of young Wistar rats. P 0.5 rats were injected intracerebroventricularly with saline (sham) or 2% autism or control serum with or without 20 nM P6. On postnatal day 26–27, rats were sacrificed and their brains were evaluated by Western blots. (**A and B**) Representative Western blots and densitometric quantification of GFAP and Iba1 expression normalized to GAPDH in the cerebral cortex of young Wistar rats. Data are presented as mean±S.E.M. based on sham (n = 6), autism serum (n = 6), autism serum+P6 (n = 6), control serum (n = 6), and control serum+P6 (n = 6). *p<0.05. ANOVA with Bonferroni’s post-hoc test and/or Student’s *t*-test.
(TIF)
