# Supplementary figures and images for: Sera from Children with Autism Induce Autistic Features Which Can Be Rescued with a CNTF Small Peptide Mimetic in Rats
Source: PLoS One. 2015 Mar 13;10(3):e0118627. doi: 10.1371/journal.pone.0118627 (PMC4359103; doi:10.1371/journal.pone.0118627)

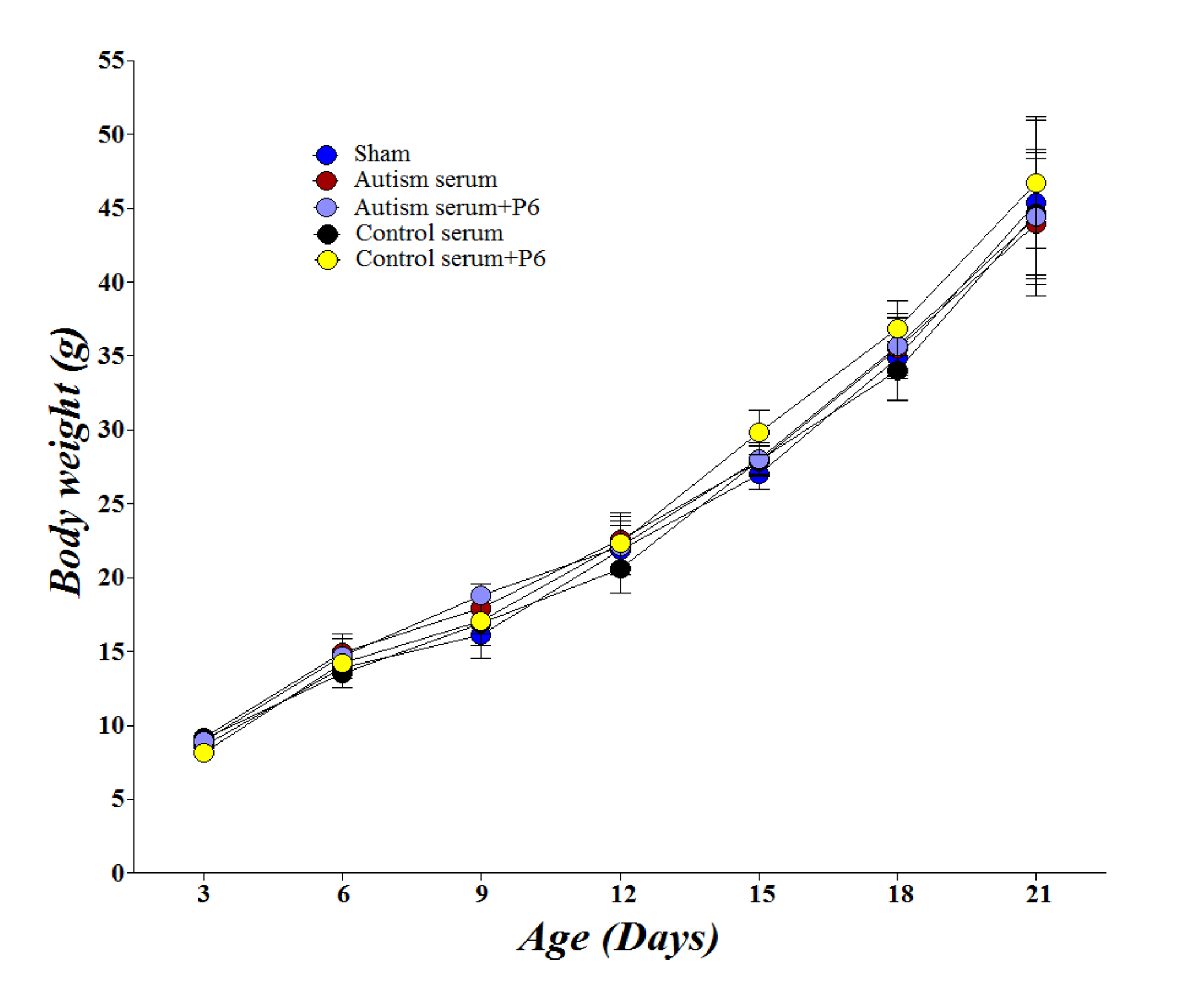

Supplement: S1 Fig — Data are presented as mean±S.E.M. based on sham (n = 17), autism serum (n = 16), autism serum+P6 (n = 17), control serum (n = 16), and control serum+P6 (n = 17). (TIF) [file pone.0118627.s001.tif]

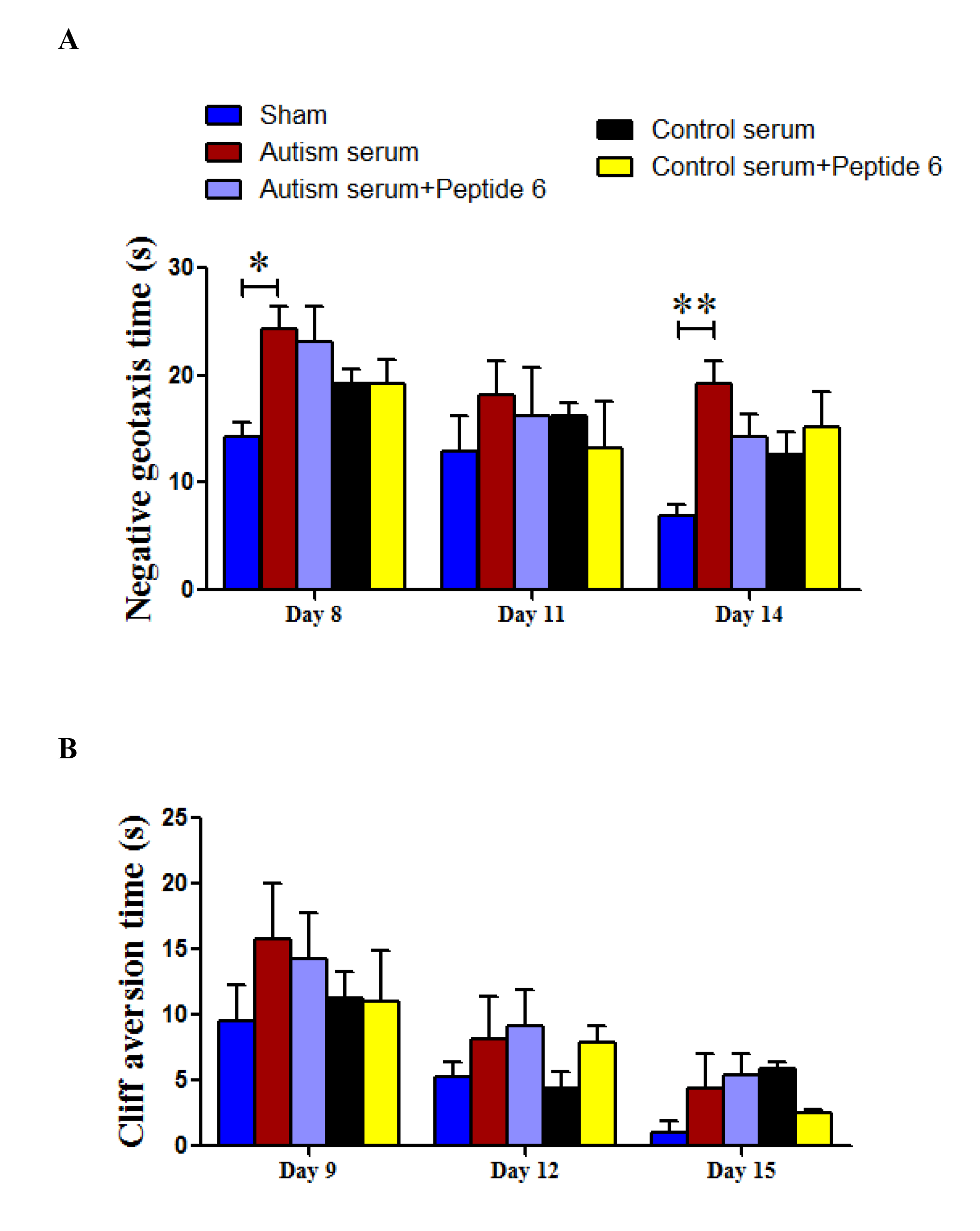

Supplement: S2 Fig — Data are presented as mean±S.E.M. based on sham (n = 17), autism serum (n = 15–16), autism serum+P6 (n = 16–17), control serum (n = 15–16), and control serum+P6 (n = 16–17). *p<0.05, **p<0.01, and ***p<0.001. ANOVA with Bonferroni’s post-hoc test and/or Student’s t-test. (TIF) [file pone.0118627.s002.tif]

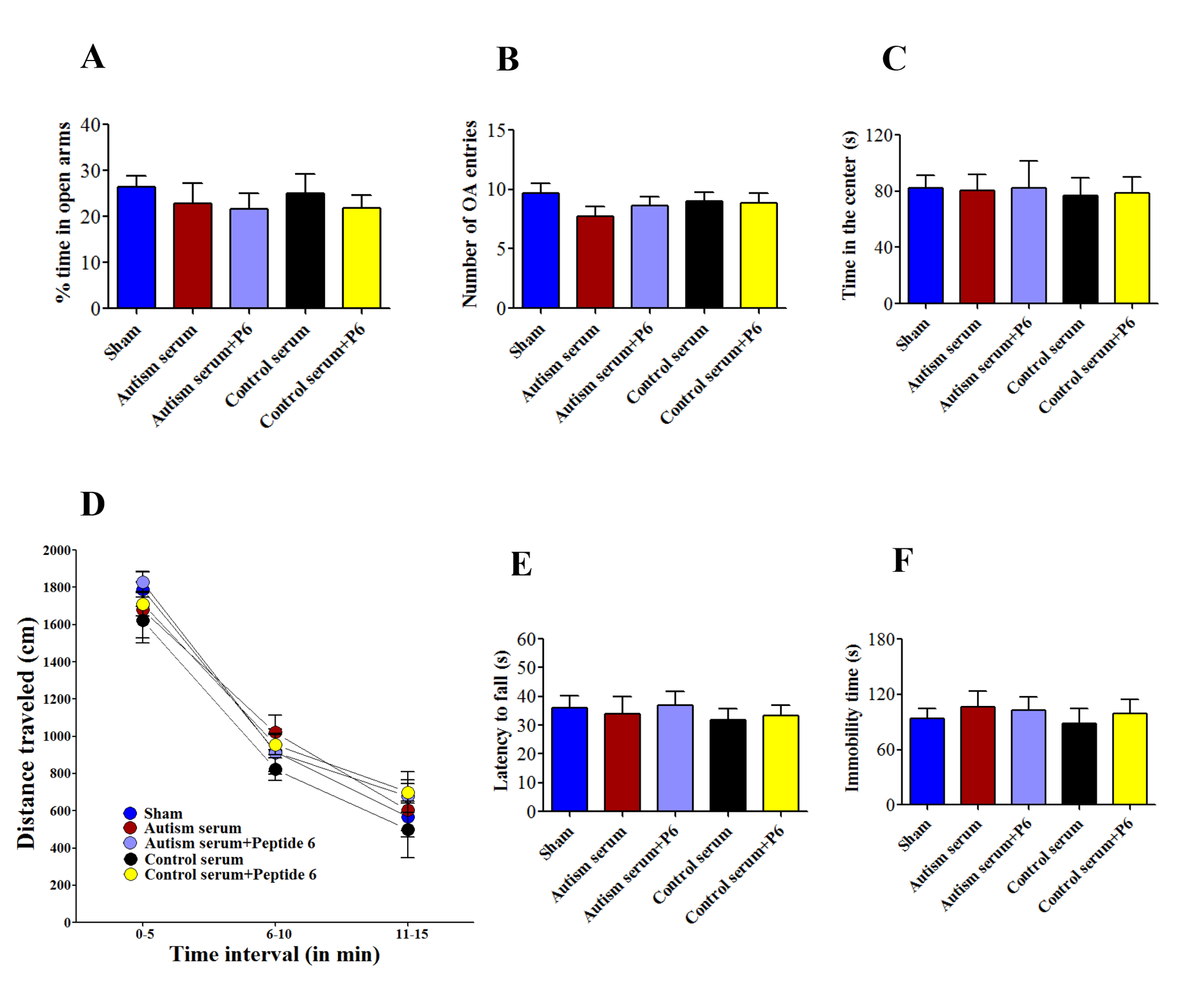

Supplement: S3 Fig — Anxiety-like behaviors were evaluated by (A) percent time in the open arm, OA (ANOVA, p = 0.8551), and (B) number of entries to OA (ANOVA, p = 0.5295) in an elevated plus maze on postnatal day 18–19, and (C) time in the center (ANOVA, p = 0.9975) in an open field arena on postnatal day 19–20. There was a trend towards decreased number of OA entries in autism serum injected young rats (sham group vs autism serum group, Bonferroni’s post hoc test, p>0.05, Student’s t-test, p = 0.08). (D) Spontaneous locomotor and exploratory activities were assessed in open field [repeated measures 2-way ANOVA, group effect, F = 0.34 (8, 219), p = 0.9513]. (E) Motor strength was evaluated by latency to fall in prehensile traction test on postnatal day 24–25 (ANOVA, p = 0.9332). (F) Behavioral despair and depression-like behavior was analyzed by immobility time in forced swim test on postnatal day 24–25 (ANOVA, p = 0.9410). Data are presented as mean±S.E.M. based on sham (n = 15–17), autism serum (n = 15–17), autism serum+P6 (n = 15–17), control serum (n = 15–17), and control serum+P6 (n = 15–17). (TIF) [file pone.0118627.s003.tif]

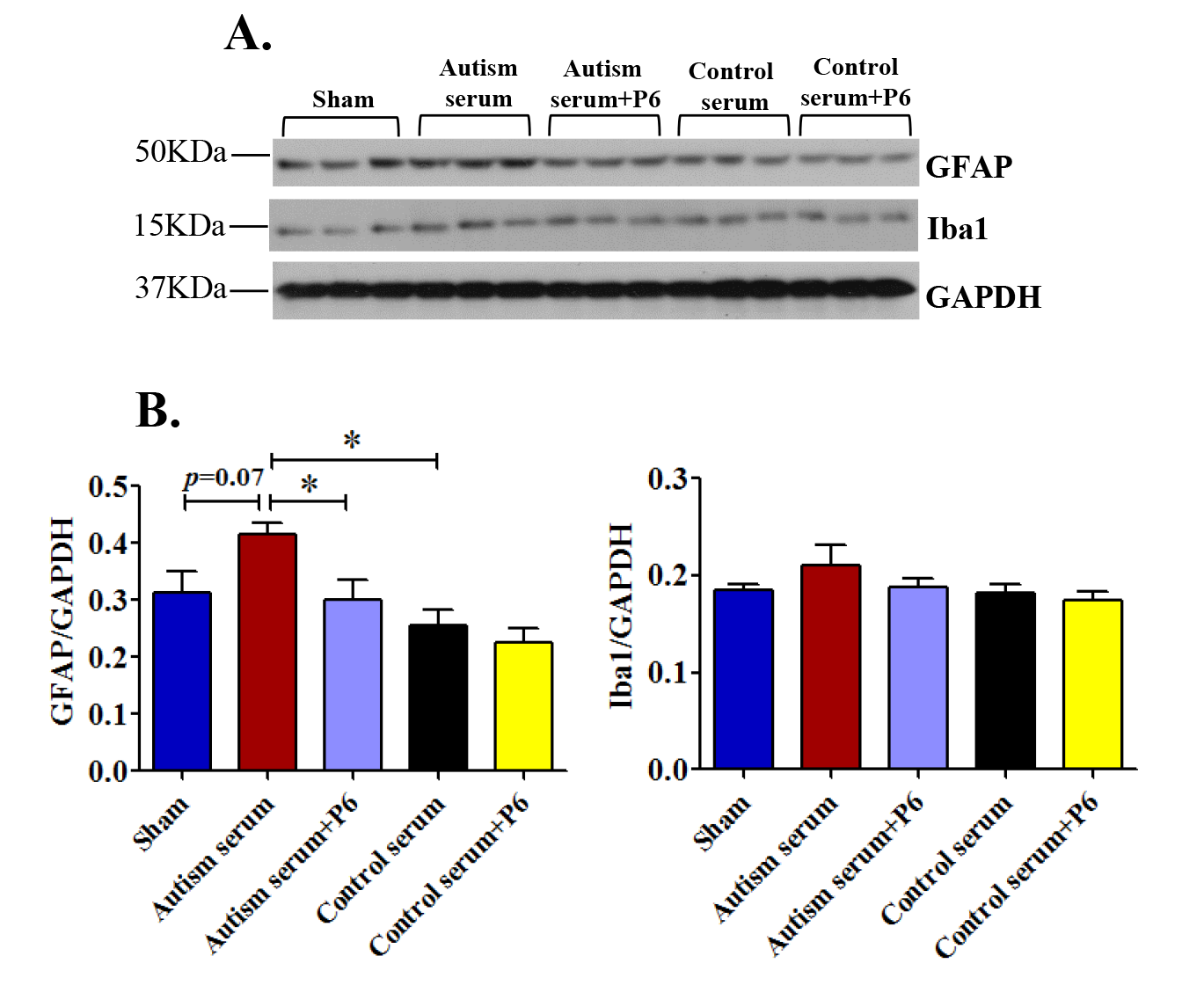

Supplement: S4 Fig — P 0.5 rats were injected intracerebroventricularly with saline (sham) or 2% autism or control serum with or without 20 nM P6. On postnatal day 26–27, rats were sacrificed and their brains were evaluated by Western blots. (A and B) Representative Western blots and densitometric quantification of GFAP and Iba1 expression normalized to GAPDH in the cerebral cortex of young Wistar rats. Data are presented as mean±S.E.M. based on sham (n = 6), autism serum (n = 6), autism serum+P6 (n = 6), control serum (n = 6), and control serum+P6 (n = 6). *p<0.05. ANOVA with Bonferroni’s post-hoc test and/or Student’s t-test. (TIF) [file pone.0118627.s004.tif]
